# Nanopriming technology for enhancing germination and starch metabolism of aged rice seeds using phytosynthesized silver nanoparticles

**DOI:** 10.1038/s41598-017-08669-5

**Published:** 2017-08-15

**Authors:** Wuttipong Mahakham, Ajit K. Sarmah, Santi Maensiri, Piyada Theerakulpisut

**Affiliations:** 1 Department of Civil and Environmental Engineering, Faculty of Engineering, The University of Auckland, Private Bag 92019, Auckland, 1142 New Zealand; 2 School of Physics, Institute of Science, Suranaree University of Technology, Nakhon, Ratchasima 30000 Thailand; 3 Salt-tolerant Rice Research Group, Department of Biology, Faculty of Science, Khon Kaen University, Khon Kaen, 40002 Thailand

## Abstract

Application of nanomaterials for agriculture is relatively new as compared to their use in biomedical and industrial sectors. In order to promote sustainable nanoagriculture, biocompatible silver nanoparticles (AgNPs) have been synthesized through green route using kaffir lime leaf extract for use as nanopriming agent for enhancing seed germination of rice aged seeds. Results of various characterization techniques showed the successful formation of AgNPs which were capped with phytochemicals present in the plant extract. Rice aged seeds primed with phytosynthesized AgNPs at 5 and 10 ppm significantly improved germination performance and seedling vigor compared to unprimed control, AgNO_3_ priming, and conventional hydropriming. Nanopriming could enhance α-amylase activity, resulting in higher soluble sugar content for supporting seedlings growth. Furthermore, nanopriming stimulated the up-regulation of aquaporin genes in germinating seeds. Meanwhile, more ROS production was observed in germinating seeds of nanopriming treatment compared to unprimed control and other priming treatments, suggesting that both ROS and aquaporins play important roles in enhancing seed germination. Different mechanisms underlying nanopriming-induced seed germination were proposed, including creation of nanopores for enhanced water uptake, rebooting ROS/antioxidant systems in seeds, generation of hydroxyl radicals for cell wall loosening, and nanocatalyst for fastening starch hydrolysis.

## Introduction

Nanotechnology has the potential to revolutionize the agriculture and play an important role in food and crop production^1, 2^. During the past decade, a number of patents and products incorporating engineered nanoparticles (NPs) into agricultural practices, e.g. nanopesticides, nanofertilizers, and nanosensors, have been developed with the collective goal to promote the efficiency and sustainability of agricultural practices requiring less input and generating less waste than conventional products and approaches^2, 3^. Silver nanoparticles (AgNPs) are the most commercialized nanomaterials widely used in antimicrobial and personal care products, building materials, water filtration, medical instruments, and in many other industrial and biomedical applications^4^. Since applications of NPs in agriculture need to be economical, ecofriendly, biocompatible and non-toxic^3, 5^, synthesis of bioengineered NPs for agricultural purpose should be compatible with these requisites. Plant based materials seem to be the best candidates for synthesizing biocompatible NPs due to their biochemical diversity of plant extract, non-toxic phytochemical constitutes, non-pathogenicity, low cost and flexibility in reaction parameters as compared to chemical synthesis methods^5, 6^.

In commercial agriculture, rapid and uniform seed germination and seedling emergence are important determinants of successful stand establishment^7, 8^. Germination begins with water uptake by the mature dry seed (imbibition) and terminates with the elongation of the embryonic axis, usually the radicle, through the seed envelope, which has as a consequence, the protrusion of the root, and later of the shoot^7^. The α-amylase is one of the key enzymes involved in degradation of starch during germination of cereal seeds and in subsequent seedling establishment^9^. This is the only enzyme which initiates hydrolysis of native starch granules and is *de novo* synthesized during the germination of cereal seeds and catalyzes the hydrolysis of α-1, 4 linked glucose polymers to release fragments that can be further broken down by other amylolytic enzymes^10^. Thus, the enhancement of α-amylase during seed germination is of interest in the field of carbohydrate research to promote economic plant growth.

All seeds stored under air dry conditions will have suffered a degree of deterioration^11^ and seeds in long-term storage will eventually lose their viability due to spontaneous biochemical damage occurring at cellular level^12^, leading to natural seed aging and subsequently limiting crop productivity. Seed priming is a technique that partially hydrates seeds in natural or synthetic compounds under specific environment to a point where germination-related metabolic processes begin, but radicle emergence does not occur^13^. Seed priming has been found to be useful for enhancing seed quality, seedling establishment and crop yields as well as increasing tolerance to environmental stresses^8, 13^. Seed priming can improve the germination of weak, damaged or aged seeds^11^ or even under adverse environment^13^. A number of commonly used priming agents include polyethelyne glycol, inorganic salts, nutrients, and plain water^12, 14, 15^. However, different priming solutions have different properties, effectiveness, and optimization of priming agents is required for each crop species^14^. Therefore, there is a growing need to develop new priming agents to enhance seed germination of various crop plants.

In recent years, several metal-based NPs (e.g., AgNPs^16^, AuNPs^5^, CuNPs^17^,^18^, FeNPs^17^, FeS_2_NPs^19^, TiO_2_NPs^20^, ZnNPs^17^,^18^, ZnONPs^21^) and carbon-based NPs (e.g., fullerene^22^ and carbon nanotubes^23^) have been applied as seed pre-treatment agents for promoting seed germination, seedling growth, and stress tolerance in some crop plants. Among these studies, only a few researchers have used seed priming strategy, in which seeds must be re-dried to their original moisture content before sowing. Thus, the mechanism behind seed nanopriming would be different from that of pre-sowing seed treatment without drying seeds. In addition, comprehensive studies on physiological and molecular mechanism of nanopriming effects on seed germination have not been elucidated, and thus there are many questions remained to be addressed, especially mechanism behind NPs-induced seed germination.

In this study, jasmine rice (*Oryza sativa* L. cv. KDML 105) was selected as a model plant since this cultivar is one of the world’s best quality aromatic rice cultivars which is in high demand for world’s markets, but it is sensitive to various environmental stresses, resulting in the reduction of yield^24^. Moreover, many seed lots of jasmine rice are naturally aged seeds due to improper storage conditions under ambient temperatures by most farmers. Therefore, the overarching aims of this study are to develop green method for the synthesis of AgNPs and present a new priming technique using phytosynthesized AgNPs to enhance the germination and starch metabolic process of rice aged seeds alternative to conventional priming. The mechanism behind AgNPs induced seed germination and starch mobilization was also proposed.

## Results

### Synthesis and characterization of phytosynthesized AgNPs

In the present study, the color of kaffir lime leaf extract changed from pale yellow to dark brown after exposure with AgNO_3_ solution for about 1 h at room temperature, indicating reduction of Ag + to Ag^0^ (Inset of Fig. S1 in Supporting information). The surface plasmon resonance (SPR) of AgNPs showed a peak centered near 443 nm at UV-vis spectra (Fig. S1), confirming the reduction of silver ions to colloidal silver.

**Figure 1:**
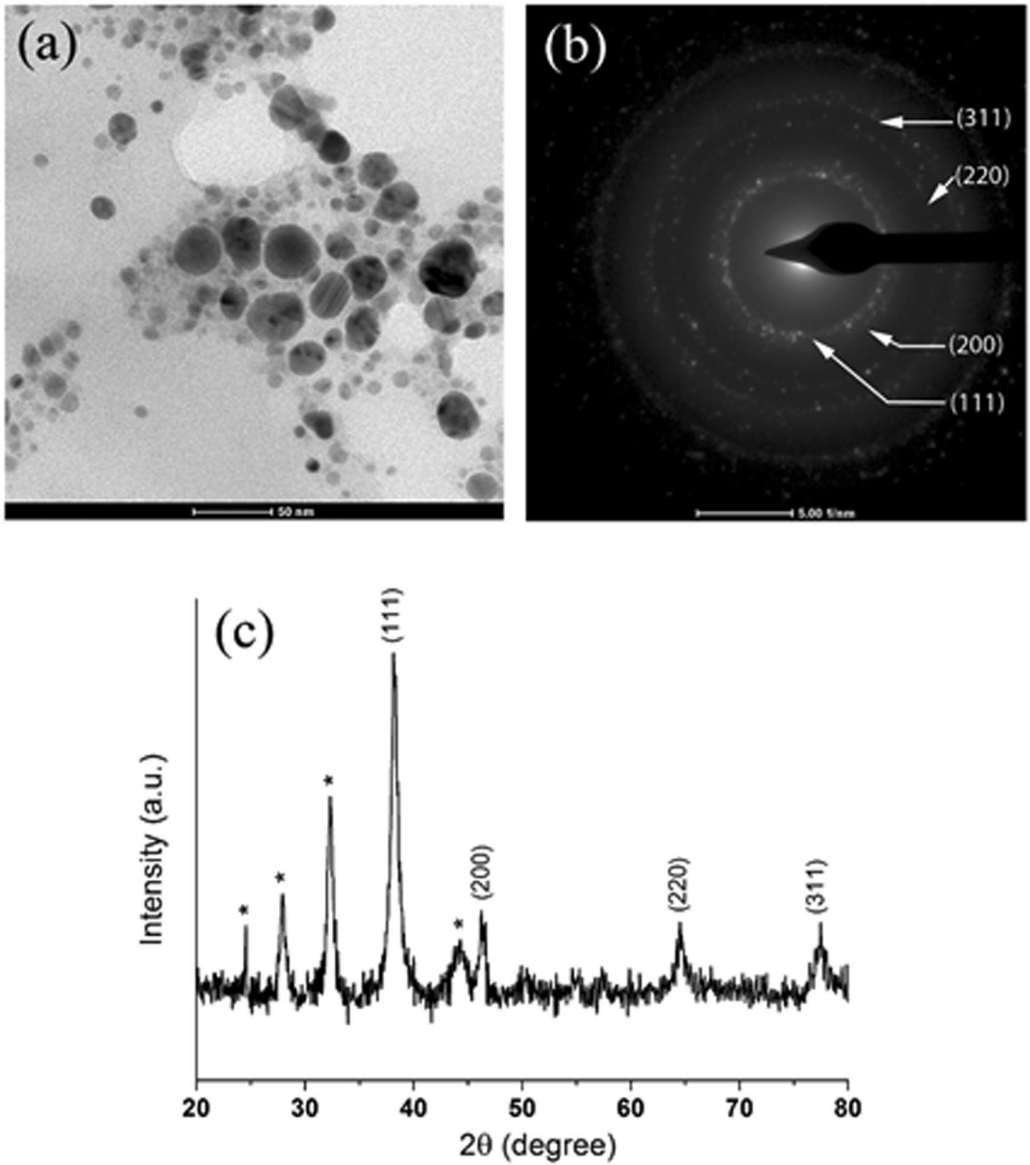
TEM image of AgNPs synthesized using kaffir lime leaf extract (scale bar, 50 nm) (**a**), SAED pattern (scale bar, 5 1/nm) (**b**) and XRD pattern (**c**).

TEM image (Fig. 1a) of the AgNPs sample shows nanoparticles exhibiting a typical spherical and an ellipsoidal morphology. The approximate particle diameter was found to be in the range of 6–36 nm. EDX analysis also confirmed the presence of elemental silver signal of the synthesized AgNPs (Fig. S2, see Supporting information). The selected area electron diffraction (SAED) pattern in Fig. 1b showed the bright circular rings, suggesting the crystalline nature of AgNPs. The diffraction rings of the AgNPs exhibited Debye-Scherrer rings assigned (111), (200), (220), and (311) lattice planes of the face centered cubic (fcc) silver, respectively, which is consistent with XRD result (Table S1 in Supporting information).

X-ray diffraction (XRD) was also used to confirm the crystalline nature of synthesized AgNPs. Figure 1c displays four prominent diffraction peaks at 2θ value of 38.2°, 44.3°, 64.6° and 77.4° indexed as (111), (200), (220) and (311) miller indices, respectively (Table S1), which are characteristic of fcc crystalline structure of metallic silver (JCPDS file No.04–0783)^25^. The unassigned peaks marked with asterisks were also observed, suggesting that the crystallization of bioorganic phase occurred on the surface of the nanoparticles. The average nanocrystalline size of synthesized AgNPs is calculated by Debye-Scherrer’s equation^25^ was found to be around 11.2 nm with respect to high intense peak (111) as shown in Table S1.

**Figure 2:**
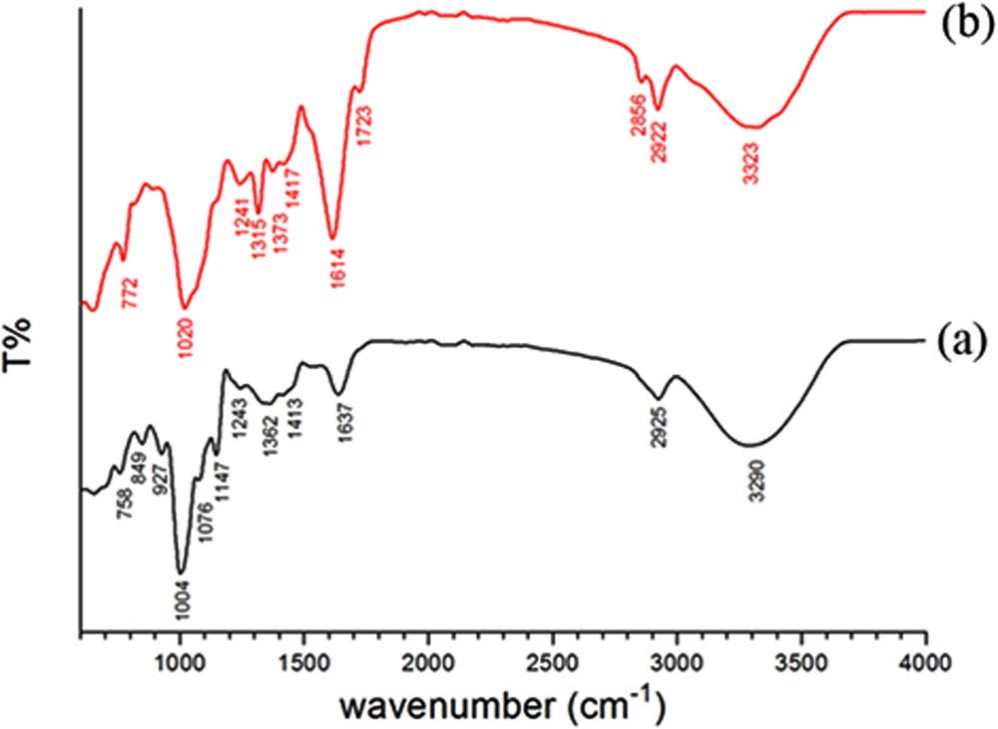
FTIR spectra of phytosynthesized AgNPs synthesized using kaffir lime leaf extract (**a**) and kaffir lime leaf extract (**b**).

FTIR analysis was conducted to identify the surface structure of the AgNPs and identify the possible functional groups (phytomolecules) of the plant extracts involved in the synthesis and stabilization of the AgNPs. In FTIR of kaffir lime leaf extracts (Fig. 2b), a number of peaks reflecting a complex nature of plant metabolites were recorded: for example, 3323 cm^−1^ (stretching vibrations of the hydroxyl (–OH) groups of alcohol and phenolic compounds)^26–28^, 2922 cm^−1^ (stretching vibrations of secondary amines in the N–H bond)^26, 29, 30^, 1614 cm^−1^ (amide I band arising due to carbonyl (–C=O) stretch of the proteins)^29, 31^, 1241 cm^−1^ (amide III band of protein)^29, 30^, and 1020 (C–N stretching vibration of aliphatic amines or to alcohols or phenols)^29^. By comparing the FTIR spectra of the plant extract and synthesized AgNPs, the band shift and intensity decrease of the hydroxyl, carbonyl and amine groups was observed (Fig. 2a), indicating that the major phytochemicals from the extract were involved in reduction and stabilization processes and coated on the AgNPs surface.

### Ag^+^ ion release in priming solution

To determine bioavailability and Ag^+^ ion release in AgNPs and AgNO_3_ priming solutions, we measured Ag^+^ concentration in the priming solutions at 1 h and 24 h after dissolution using ICP-OES technique. At 1 h, the release of Ag^+^ ions in both concentrations of AgNO_3_ priming solutions (10 and 20 mgL^−1^) was significantly higher than those of AgNPs priming solutions (Table S2), suggesting the rapid release of Ag^+^ ions in AgNO_3_. At 24 h of dissolution, Ag^+^ ion release in both AgNO_3_10 and AgNO_3_20 drastically increased with concentration change of about 1.12–6.7 times compared to their respective solutions at 1 h of dissolution. In contrast, Ag^+^ ion release in both AgNPs10 and AgNPs20 solution slowly increased with concentration change of about 1.2–1.5 times compared to their respective solution at 1 h of dissolution. At 24 h, the Ag^+^ concentrations in AgNO_3_10 and AgNO_3_20 solutions were 12.7–21.6 and 25.5–43.5 times higher than AgNPs10 and AgNPs20 priming solution, respectively. No Ag^+^ concentration was observed from deionized water used as hydropriming solution.

### AgNPs could mediate hydroxyl radicals as determined by Electron Spin Resonance (ESR) analysis

**Figure 3:**
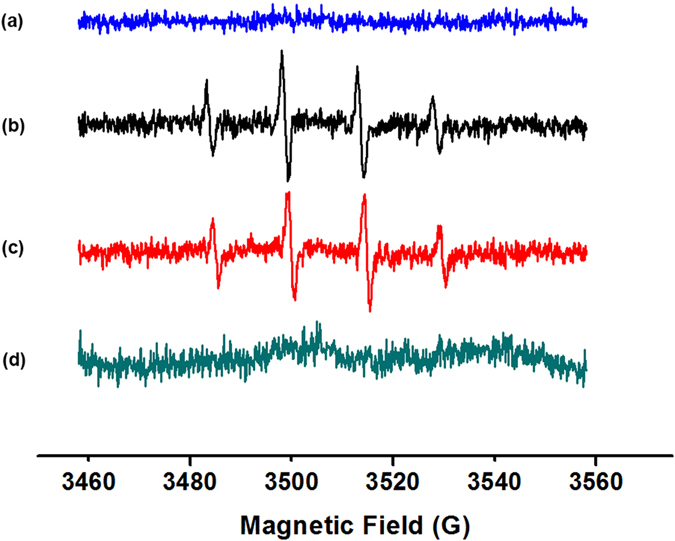
Electron spin resonance (ESR) analysis of hydroxyl radicals generated by AgNPs and AgNO_3_. ESR spectra of (**a**) control sample containing 50 mM DMPO + 0.5 mM H_2_O_2_ + 10 mM pH 3.6 HAc-NaAc in the absence of AgNPs or AgNPs, (**b**) standard Fenton reaction (50 mM DMPO + 0.5 mM H_2_O_2_ + 2.5 mM FeSO_4_), (**c**) sample containing 50 mM DMPO + 0.5 mM H_2_O_2_ + 10 mM pH 3.6 HAc-NaAc in the presence of 10 mg L^−1^ AgNPs, and (**d**) sample containing 50 mM DMPO + 0.5 mM H_2_O_2_ + 10 mM pH 3.6 HAc-NaAc in the presence of 10 mg L^−1^ AgNO_3_.

Hydroxyl radical (•OH) generation in the priming solution was analyzed using ESR, which is the most accurate and sensitive technique for the detection of free radicals. The spin trap DMPO, which reacts covalently with •OH, gives rise to stable secondary radical adduct that can be detected at room temperature using ESR. A four line spectrum with a 1:2:2: intensity ratio is of indicative of the formation of DMPO-OH spin adduct. ESR analysis indicated that AgNPs were able to generate •OH compared with standard Fenton reaction (Fig. 3). In contrast, deionized water and AgNO_3_ did not produce ESR signal under the same conditions, indicating that Ag^+^ did not produce •OH.

### Acceleration of seed germination and seedling growth by nanopriming

**Figure 4:**
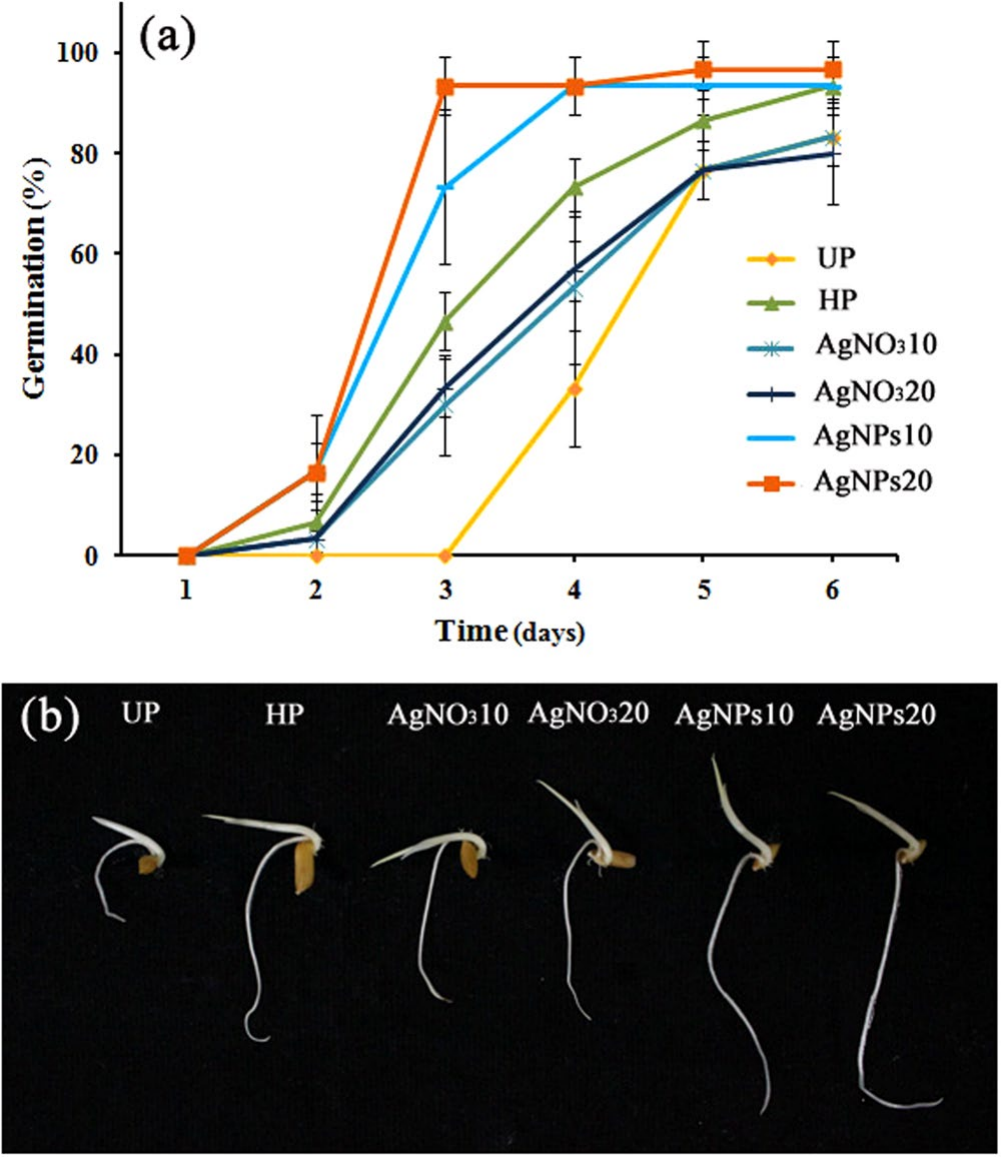
Germination rate of rice seeds after priming with different priming agents (**a**) and phenotype of rice seedlings after 6 day germination (**b**). Data are presented as means of three replicates containing 10 seeds each ± standard error of means. (Abbreviations of each treatment are defined in materials and methods).

As shown in Fig. 4a, priming the rice seeds with nanopriming solutions (AgNPs10 and AgNPs20) could accelerate the early seed germination and germination percentage compared to those of the unprimed and other primed groups (*p* < 0.05). For example, while the control seeds germinated on the fourth day after incubation, 16.6% of both nanoprimed seeds were able to germinate during the second day after incubation (*p* < 0.05). Moreover, both nanoprimed seeds had reached 93% of germination on the fourth day after incubation. A significant increase of seed germination was observed for nanoprimed seeds on the third day. At this time, the germination percentage for seeds that were primed with AgNPs10 and AgNPs20 averaged 73.3% and 93.3%, respectively, while seed germination percentage of the hydropriming, AgNO_3_10 and AgNO_3_20 treatments averaged 46.6, 30.0 and 33.3%, respectively (*p* < 0.05). The germination percentage of nanoprimed seeds was significantly higher than that of both groups of AgNO_3_-primed seeds, but was not significantly different from hydropriming group (Table 1).

**Table 1:** Impact of different priming agents on germination percentage, germination index, germination rate, vigor index and seed water uptake. Means, in each column, followed by similar letter are not significantly different at the 5% probability level-using Duncańs Multiple Range Test.

Treatment	Germination (%)	Germination index	Germination rate	Vigor Index	Seed water uptake (%)	
					4 h	24 h
UP	83.3bc	3.8d	1.8d	207.7c	9.1c	14.8c
HP	93.3ab	7.0b	2.7b	484.7b	13.2b	17.7b
AgNO_3_10	83.3bc	5.4c	2.2c	267.8c	10.3c	15.4c
AgNO_3_20	80.0c	5.6c	2.2c	258.0c	10.1c	15.1c
AgNPs10	93.3ab	9.0a	3.2a	600.5a	15.6a	19.1a
AgNPs20	96.6ab	9.9a	3.5a	631.0a	16.6a	19.3a

In addition, the germination index (GI) and germination rate (GR) of the tested seeds were also calculated, and it was found that nanopriming treatment greatly increased GI and GR values compared to those of unprimed and other primed seeds (Table 1). The phenotypic characteristics of the rice seedlings developed from all nanoprimed seeds are normal without the sign of toxicity (Fig. 4b). After 6 days of germination, nanopriming significantly increased vigor index compared to unprimed control and other priming treatment (Table 1). Also, priming with nanosolution significantly increased the lengths of roots and shoots and seedling biomass (mg weight) compared to the unprimed and other primed groups (*p* < 0.05) (Fig. S3a–d). Overall, priming the aged seeds of jasmine rice with phytosynthesized AgNPs at optimum doses (10 and 20 mgL^−1^) increased seed germination, vigor strength and seedling growth.

### Seed water uptake

To test whether different priming agents can affect seed imbibition at the early germination process, seed water uptake at 4 and 24 h of imbibition was determined. It was found that all primed seeds adsorbed water faster than those unprimed at the initial (4 h) and later (24 h) periods of imbibitions (*p* < 0.05) (Table 1). Nanoprimed seeds (both AgNPs10 and AgNPs20) imbibed water faster than other primed groups at the very beginning (4 h) of imbibition (*p* < 0.05). At 24 h, seed water uptake of nanopriming treatment was still higher than hydropriming and AgNO_3_ priming treatments.

### Internalization of Ag in rice seeds

Internalization and accumulation of silver (Ag) of primed seeds were analyzed by ICP-OES. As shown in Table S3, no Ag was detected in unprimed and hydroprimed seeds (detection limit of Ag analysis by ICP-OES is 0.001 ppm), indicating that the Ag element is not present in rice seeds. On the contrary, both groups of seeds treated with AgNO_3_ or AgNPs priming solutions showed the accumulation of Ag, albeit very low concentration. This implied that Ag^+^ ions released from AgNO_3_ or AgNPs solutions w﻿ere﻿ able to penetrate the seed coat into endosperm and embryonic tissues. The concentration of Ag increased as the external concentration of AgNPs or AgNO_3_ increased (Table S3). Ag concentrations of AgNPs10-primed seeds were significantly lower than both groups of AgNO_3_ treatments, while those of AgNO_3_20-primed seeds were the highest. Although Ag concentrations of AgNPs20 were significantly lower than AgNO_3_20, they were not statistically different from AgNO_3_10. The higher concentrations of Ag found in AgNO_3_20-primed seeds could be due to higher solubility and bioavailability of AgNO_3_ solution compared to those of AgNPs solution. This result corresponded well with the data on Ag^+^ ion release (Table S2), demonstrating that AgNO_3_ priming solution had higher Ag^+^ concentration than AgNPs priming solution.

### Nanopriming can enhance starch metabolism for rice seedling growth

**Figure 5:**
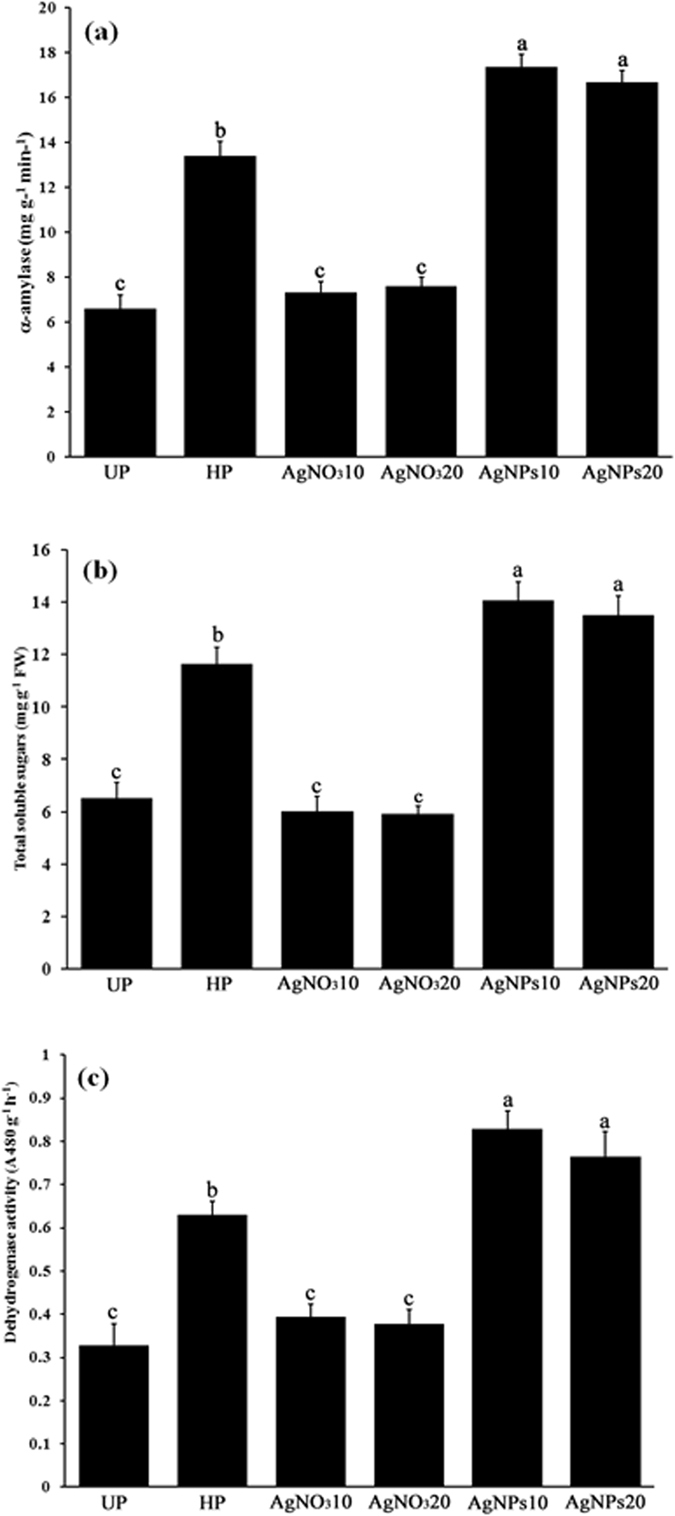
Impacts of different priming agents on starch metabolism and root dehydrogenase activity of rice seedlings, (**a**) α-amylase activity, (**b**) total soluble sugars, and (**c**) dehydrogenase activity. Data are presented as mean ± SE. Means denoted by the different letter are significantly different at *p*  <0.05 according to the Duncan’s multiple range test.

Starch metabolism in rice seedlings was assessed in terms of α -amylase activity and total soluble sugars. After 6 days of seed germination, nanopriming treatments significantly enhanced α-amylase activity in rice seedlings compared with control and other primed seedlings (Fig. 5a). The α-amylase activity of seedlings increased 2.6 and 2.5 folds in AgNPs10 and AgNPs20 priming treatments, respectively, compared to control seedings. The α-amylase activities of seedlings of AgNPs10 and AgNPs20 treatments were almost 1.3 and 1.2 times greater than those in the hydropriming group, while the α-amylase activities in the AgNO_3_10 and AgNO_3_20-treated seedlings were not significantly different from the control. Likewise, the total soluble sugar contents of seedlings were induced from 2.0 to 2.2 folds by nanopriming treatments (AgNPs10 and AgNPs20) compared to the control, while 1.8-fold increase was observed in the hydroprimed seedlings (Fig. 5b). In the AgNO_3_ priming treatments, the total soluble sugars remained almost unchanged compared to control.

We also confirmed above results by measuring amylase production and secretion from embryoless half-seeds using a starch agar plate α-amylase assay method. Clear zones appeared after IKI staining when isolated endosperms of half seeds were incubated in starch agar containing gibberellins (GA), indicating expression and secretion of α-amylases that hydrolyzed starch^32^. After 3 days of incubation, numbers of clear zone recorded from nanoprimed seeds were higher than that from other primed seeds and unprimed control (Fig. S4), corresponding with rapid germination of nanoprimed seeds compared to other treatments. Besides, clear zone areas observed in nanoprimed seeds were larger than in other priming treatments, indicating the higher levels of α-amylase expression and production from nanoprimed seeds. Control plate without GA showed no amylase production from embryoless half-seeds.

### Starch granule morphology

**Figure 6:**
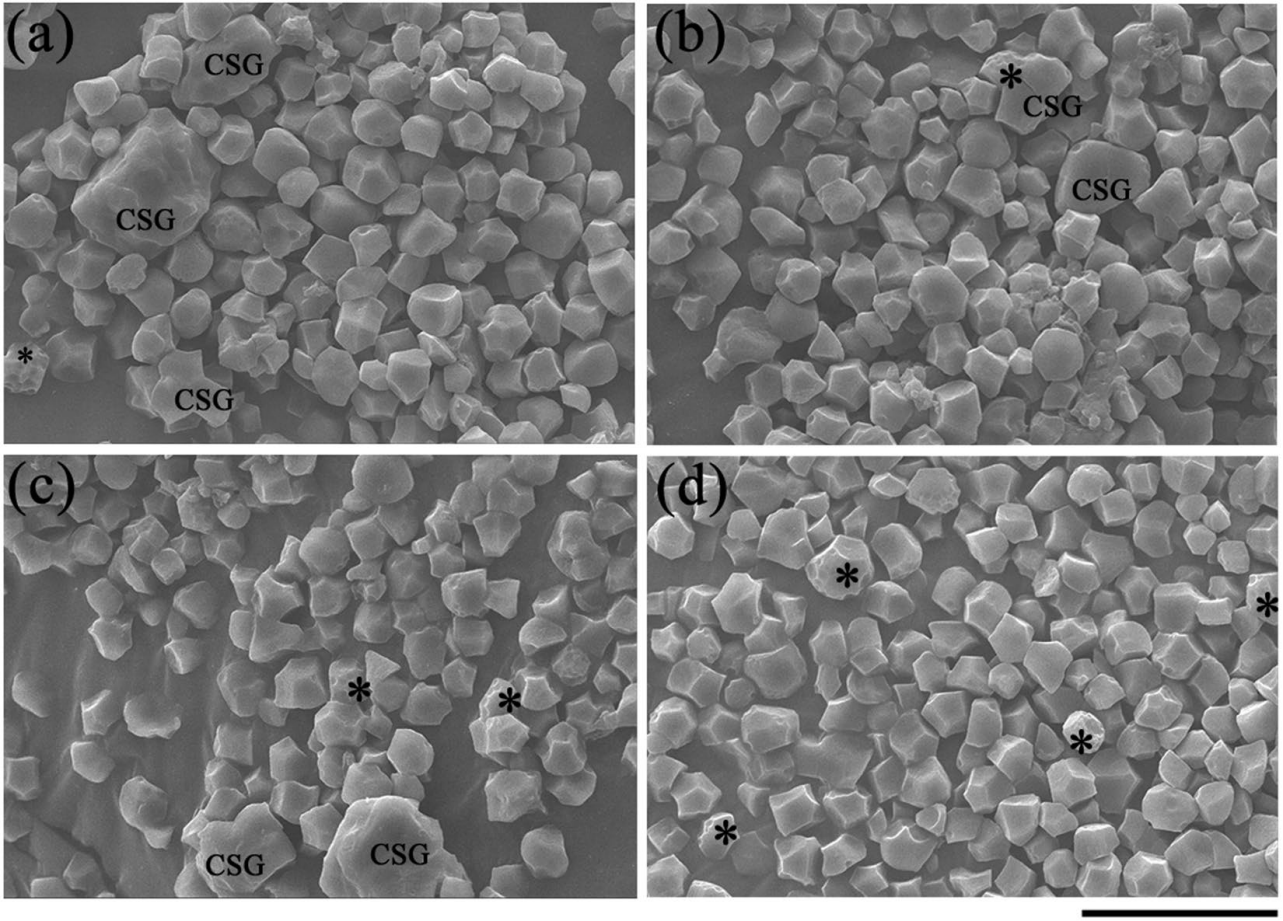
SEM micrographs of starch granules of unprimed seeds (**a**), AgNO_3_-primed seeds (**b**), hydroprimed seeds (**c**), and AgNPs-primed seeds (**d**). Starch granules were isolated from seeds after 24 h of imbibition. Asterisk indicates starch granule with small pits. CSGs, compound starch granules. (Scale bar = 20 µm).

We also observed the morphological change of isolated starch in both unprimed and primed seeds after 24 h of imbibition. It can be observed from SEM results that in general individual starch granules (ISGs) of rice seeds of unprimed and primed groups were polyhedral in appearance with irregular shapes (Fig. 6), corresponding with previous reports^33, 34^. Some compound starch granules (CSGs) were observed in control, hydroprimed and SN-primed seeds, suggesting that starchy endosperm of these treatments had low starch decomposition rate at initial phase of germination. On the contrary, starch granules of AgNPs-primed seeds did not form the CSGs and contained some smaller ISGs, indicating higher degrees of starch decomposition. Moreover, some starch granules of nanoprimed seeds had small pits on their surface, indicating a higher amylase activity compared to control and other primed seeds.

### Nanopriming enhanced dehydrogenase activity in rice seedlings

Dehydrogenase activity is an indicator of root vitality and used as comprehensive assessment index reflecting the metabolic activity level and root’s ability to absorb nutrients and water^35^. After 6 days of seed germination, pronounced increase (∼2.3 to 2.5-fold) in root dehydrogenase activity over unprimed seedlings was found in nanopriming treatments (Fig. 5c). Root dehydrogenase activity of AgNPs10 and AgNPs20 priming treatments was also significantly higher (∼1.3- and 1.2-fold) than that of the hydropriming group, while in the silver nitrate treatments it remained almost unchanged compared to the control.

### Nanopriming enhanced aquaporin gene expression in aged rice seeds

In order to elucidate the molecular basis for changes in seed metabolism in response to priming treatments, the expression levels of two aquaporin genes, *PIP1;1* and *PIP2;1*, which are responsible for water uptake were monitored in germinating seeds at 24 h of imbibition. The expression levels of the two aquaporin genes in AgNO_3_-primed seeds and unprimed control were not obviously different. On the other hand, priming with AgNPs markedly enhanced the expression of aquaporin genes, especially *PIP2;1* as compared to the control and other priming treatments (Fig.S5), coinciding with the higher percentage of seed water uptake at 24 h (Table 1). The transcript levels of both *PIP1;1* and *PIP2;11* in hydroprimed seeds also increased as compared with AgNO_3_-primed seeds and control, but their expression levels were lower level than nanopriming treatments.

### Nanopriming modulated antioxidant enzyme activity in seeds after priming

**Figure 7:**
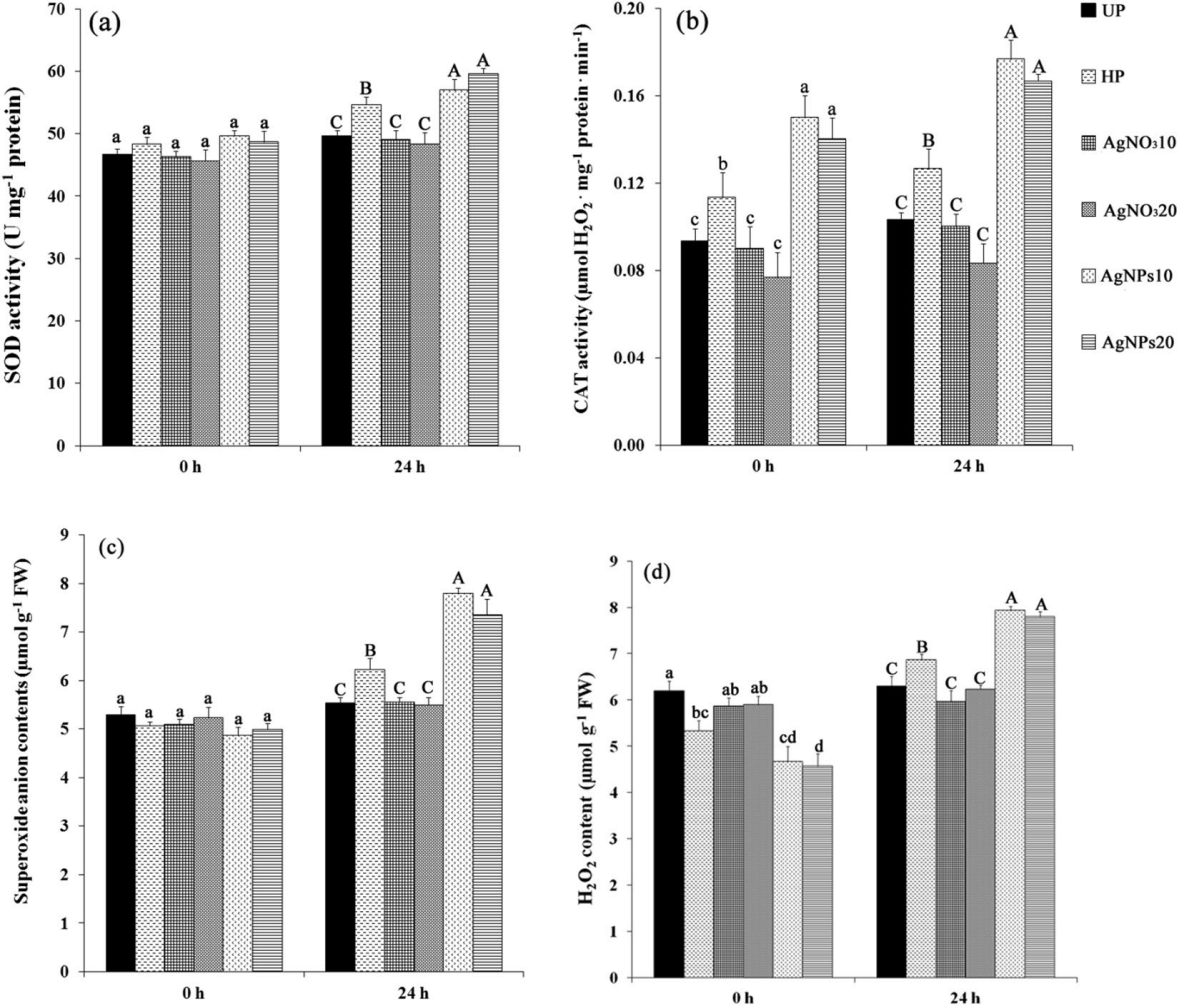
Antioxidant enzyme activities and ROS contents in rice seeds of different priming treatments compared with unprimed control. After priming and seeds were re-dried, activities of SOD (**a**), CAT (**b**), and contents of O_2_^•–^ (**c**) and H_2_O_2_ (**d**) were determined at 0 h and 24 h of imbibition. Different letters denote significant difference at *p* ≤ 0.05 according to the one-way ANOVA with Duncan’s multiple range test.

Antioxidant enzymes (SOD and CAT) activities in unprimed and primed seeds varied significantly ( *p* < 0.05) in response to different seed priming treatments (Fig. 7a,b). In dry seeds (0 h of imbibition), SOD activities of both unprimed control and primed seeds were on the same level (Fig. 7a). However, when seeds were imbibed in water for 24 h, activity levels of SOD in both nanoprimed seeds and hydroprimed seeds were higher than those in the unprimed control and AgNO_3_-primed seeds, although the values of AgNPs10-primed seeds were not significantly different from hydroprimed seeds. Interestingly, considerable increase in CAT activity in nanoprimed seeds was observed in dry seeds (0 h of imbibition) after priming with AgNPs compared to control and other primed seeds (Fig. 7b). After 24 h of imbibition, activity levels of CAT in AgNPs10- and AgNPs20-primed seeds increased by 71% and 61%, respectively, compared with the control, which coincides with the rapid radicle protrusion and confirms the connection between the raise in CAT activity and radicle protrusion. Hydroprimed seeds also had higher CAT activity compared to control and AgNO_3_-primed seeds, which had lowest CAT activities, corresponding with their delay in germination (Fig. 4a).

### Quantification of reactive oxygen species (ROS) in unprimed and primed seeds

The contents of two different ROS species, i.e. hydrogen peroxide (H_2_O_2_) and superoxide anions (O_2_^•–^) were quantified spectrophotometrically in the embryos of seeds at 0 and 24 h. At 0 h (dry seeds), the O_2_^•–^ contents in the embryos of all tested seeds were not significantly different (Fig. 7c). In contrast, when seeds were imbibed in water for 24 h, O_2_^•–^ contents of both AgNPs10- and AgNPs20-primed seeds significantly increased by ∼42% and 33%, respectively compared to control. The contents of O_2_^•–^ in hydroprimed seeds also slightly increased (∼12%) compared to control, while there were no significant changes of O_2_^•–^ between AgNO_3_ treatments and control. Interestingly, priming treatments with AgNPs can reduce H_2_O_2_ contents as both nanoprimed seeds showed lower levels than control and AgNO_3_-primed seeds at 0 h of imbibition (Fig. 7d), whereas AgNPs10 showed no significant difference from hydroprimed seeds. At 24 h of imbibition, H_2_O_2_ contents of all tested seeds leveled up. Interestingly, H_2_O_2_ contents of AgNPs10- and AgNPs20-primed seeds increased by ∼33% and 32%, respectively, compared to control. Also, levels of H_2_O_2_ in nanoprimed seeds were higher than hydroprimed and AgNO3-primed seeds. Hydroprimed seeds showed a slight increase in H_2_O_2_ contents (∼15%) compared to control, whereas no significant differences between unprimed control and AgNO_3_-treated seeds were observed.

### *in vivo* fluorescence imaging and histochemical localization of ROS production

**Figure 8:**
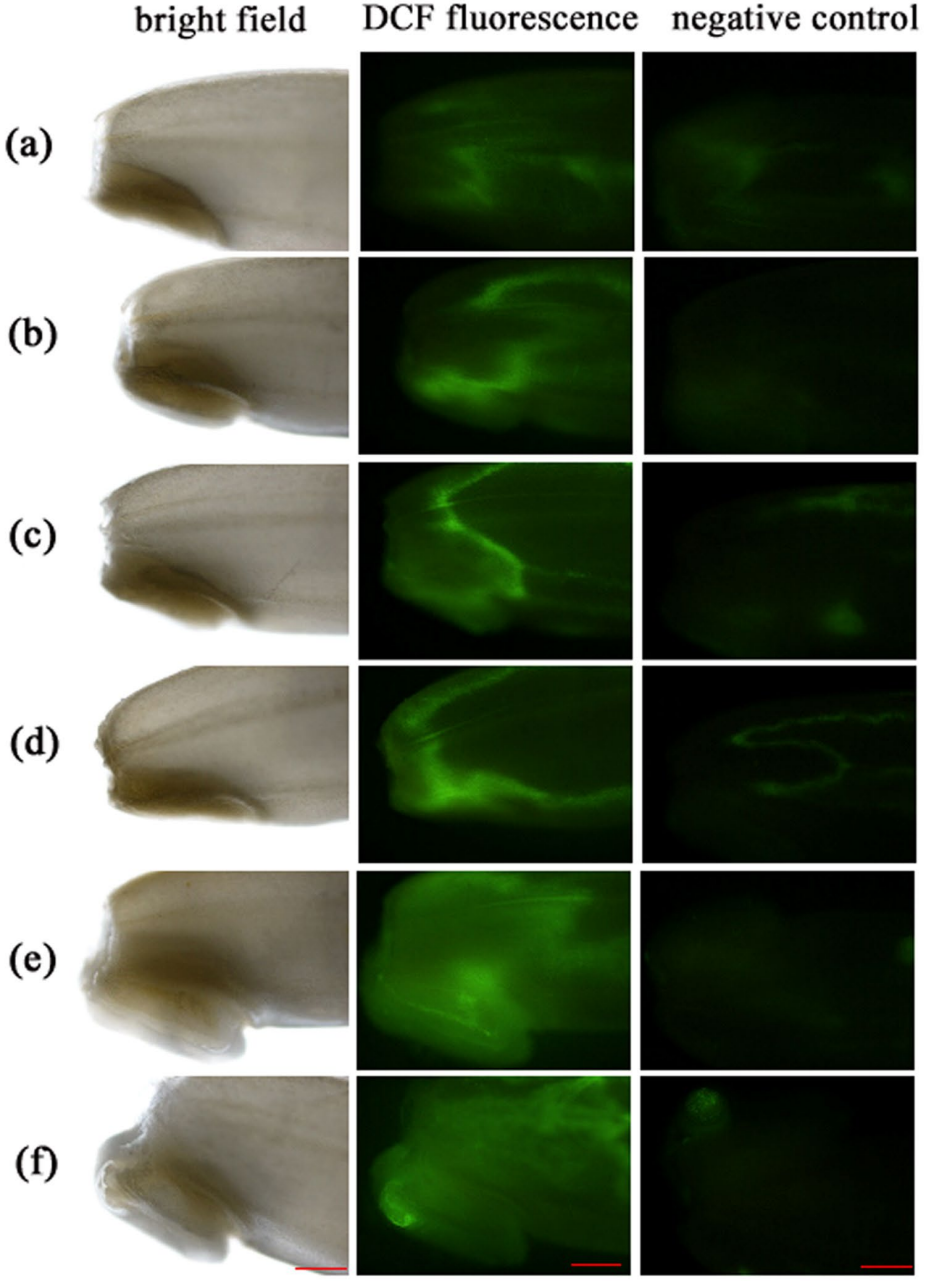
Localization of H_2_O_2_ of rice seeds imbibed for 24 h using *in vivo* fluorescence imaging technique. Seeds imbibed at 24 h were loaded with DCF-DA fluorescence probe for 30 min and subjected to fluorescence microscopy, while seeds pre-incubated with ascorbate (peroxide scavenger) before loading with fluorescence probe were used as negative control. Bright filed images were also recorded. Representative images of imbibed seeds of (**a**) unprimed control, (**b**) hydropriming, (**c**) AgNO_3_10 priming, (**d**) AgNO_3_20 priming, (**e**) AgNPs10 priming, and (**f**) AgNPs20 priming treatments were presented. (Scale bar = 20 µm).

The distribution and accumulation of H_2_O_2_ in germinating seeds at 24 h were visualized with a specific fluorescent probe, DCF-DA. After 24 h of imbibition, coleorhiza of both hydroprimed and nanoprimed seeds began to protrude, while AgNPs-primed seeds and unprimed control did not. The fluorescence signal intensities for nanoprimed and hydroprimed treatments were stronger than those of the unprimed and AgNO_3_-primed treatments, whereas the H_2_O_2_-excited fluorescence intensity was stronger in nanoprimed seeds than hydroprimed seeds (Fig. 8). Fluorescence imaging also showed that H_2_O_2_ was located mainly in coleorhizal zone and partly in the aleurone layer in germinating seeds.

**Figure 9:**
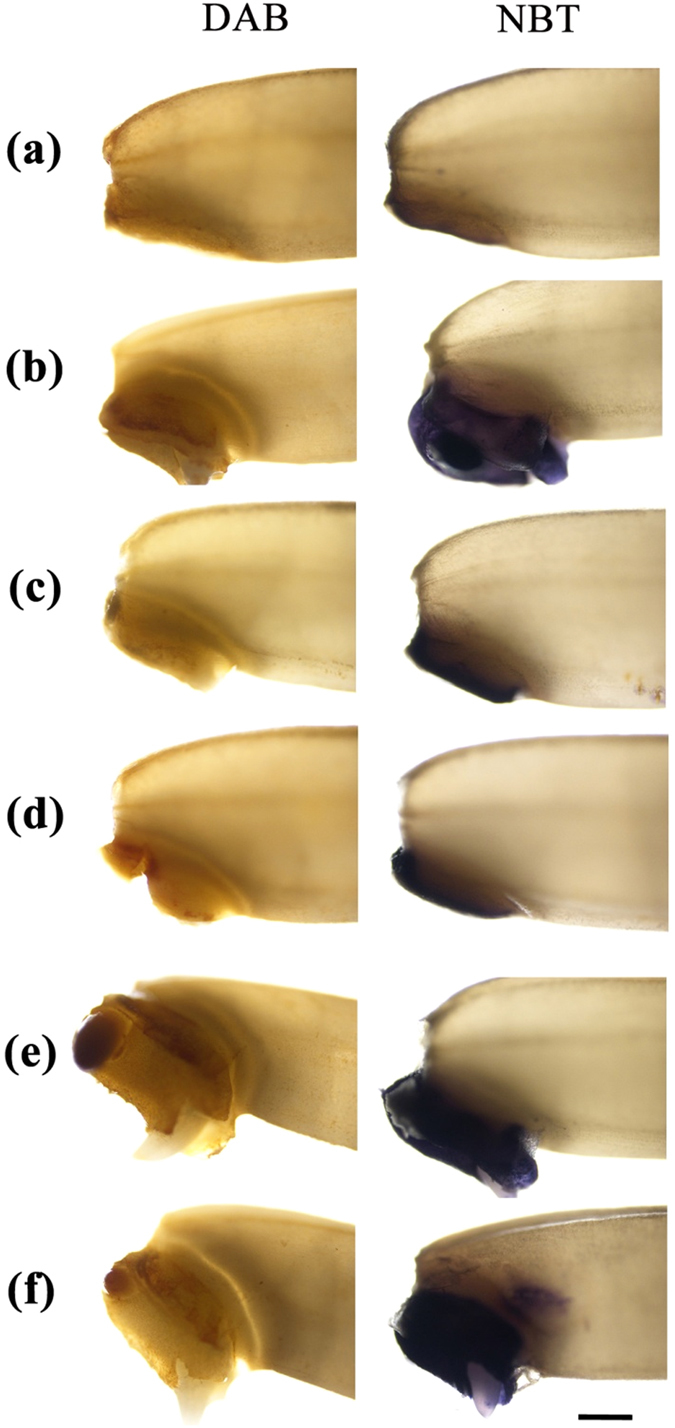
Histochemical localization of H_2_O_2_ and O_2_^•–^ in rice seeds imbibed for 36 h using DAB (**a**) and NBT (**b**) staining, respectively. Representative bright field images of imbibed seeds of (**a**) unprimed control, (**b**) hydropriming, (**c**) AgNO_3_10 priming, (**d**) AgNO_3_20 priming, (**e**) AgNPs10 priming, and (**f**) AgNPs20 priming treatments were presented. (Scale bar = 20 µm).

After 36 h of imbibitions, we localized H_2_O_2_ and O_2_^•–^ in germinating seeds using DAB and NBT staining, respectively. Results showed that in nanoprimed and hydroprimed seeds embryo and aleurone layer were somewhat stained by DAB, whereas embryo showed stronger staining than aleurone layer. Coleorhiza and radicle of nanoprimed seeds, which were more developed than unprimed and other primed seeds, showed higher accumulation of brown spots indicating the higher level of H_2_O_2_. Besides, the production and accumulation of H_2_O_2_ were greater in coleorhiza and radicle than in coleoptiles of germinating seeds (Fig. 9). Also, NBT staining indicated a marked increase of O_2_^•–^ in germinating nanoprimed seed than unprimed control and other primed seeds (Fig. 9). Although NBT stained strongly as dark purple color product in coleorhiza and radicle of nanoprimed seeds, it also stained coleoptiles with pale purple, indicating O_2_^•–^ could participate in cell elongation in these structures during germination.

## Discussion

### Characterization of phytosynthesized AgNPs

Characterization of photosynthesized AgNPs revealed some useful information. For example, reduction of Ag^+^ ions into AgNPs during exposure to the plant extracts could be visualized by color change, with kaffir lime leaf extracts turning from pale yellow to dark brown color (Fig. S1), indicating the reduction of Ag^+^ ions into AgNPs. Change in color was due to the excitation of the surface plasmon resonance (SPR) in AgNPs^36^. UV-vis absorption spectrophotometer is used to investigate the SPR phenomenon. In this study, a single, strong, and broad SPR peak at 441 nm was observed in the UV-vis spectrum, confirming the successful synthesis of AgNPs. Previous studies suggested that a SPR peak located between 410 and 450 nm exists for AgNPs and could be attributed to spherical shaped NPs^36^. The SPR peak is reported to be very sensitive to the size and shape of the NPs, amount of plant extract, AgNO_3_ concentration, and the type of phytochemicals or biomolecules present in the leaf extract^37^. Broadening of UV–visible peak observed in this study also suggested that phytosynthesized AgNPs were polydisperse in nature^38^.

TEM micrographs of the phytosynthesized AgNPs indicated that the particles were spherical and ellipsoidal, having an average size of 6–36 nm (Fig. 1a). SAED pattern with bright circular rings indicates the crystalline nature of AgNPs, which are similar to previous report^26^. Our SAED data were also corroborated with the XRD pattern of the same materials (Fig. 1c). XRD pattern of phytosynthesized AgNPs matches with the characteristics of fcc crystalline structure of metallic silver (JCPDS file No.04–0783)^25^. Also, the intensity and broadening of peaks lend further support that the obtained products are crystalline in nature and small crystallite size, respectively. The unassigned peaks marked with asterisks could be due to the crystallization of bio-organic phase, e.g. polysaccharides and proteins present in the leaf extracts, occurring on the surface of AgNPs^31^.

### Possible mechanism of AgNPs formation

**Figure 10:**
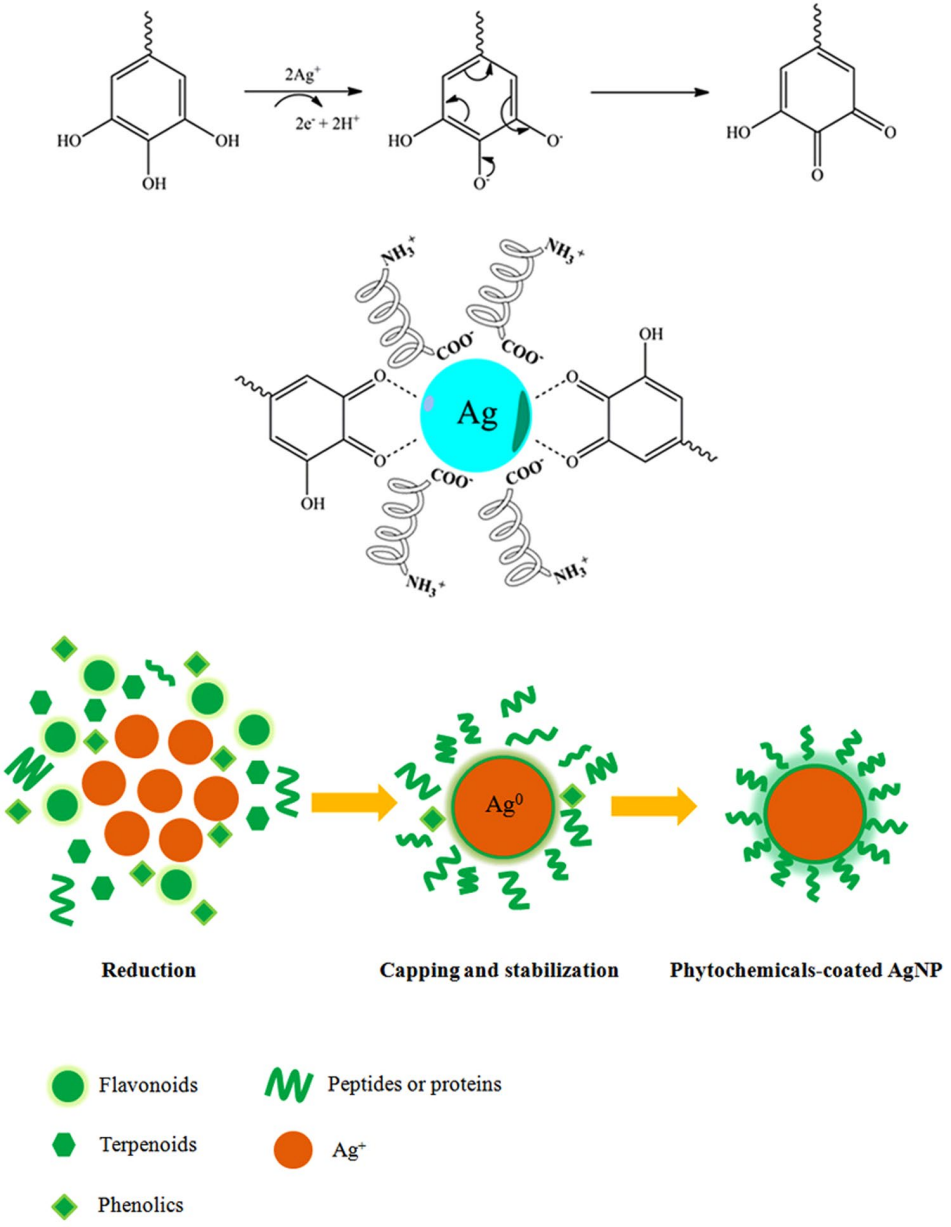
The plausible mechanism of the formation of AgNPs mediated by phytochemicals from kaffir lime leaf extract.

Our FTIR analysis showed various functional groups of phytochemicals found in kaffir lime leaf extract coated onto AgNPs surface, especially hydroxyl, carbonyl and amine groups. These functional groups could be derived from flavonoids, phenolic compounds, carbohydrates, sugars and proteins, which could act as reducing and stabilizing agents^39^. Kaffir lime leaves have been reported to have high levels of natural antioxidant polyphenols, including flavonoids and tannins, terpenoids, and volatile compounds, e.g. citronellal monoterpenoid (linalool), acyclic terpene alcohol and viridiflorol^40^. Based on the phytochemicals abundant in the plant leaves, the plausible mechanism of AgNPs formation using phytochemicals is shown in Fig. 10. Upon the addition of plant extracts to the Ag^+^ aqueous solution, the Ag^+^-phytomolecule complex intermediate is formed, and the phenolic groups could donate electron or H^+^ to Ag^+^ ions resulting in the reduced Ag^0^. After the phenol ring undergoes oxidation, it converts to its quinone form. The electrochemical potential difference between Ag^+^ ions and phytomolecules drives the reaction, and the formed AgNPs are stabilized by the lone pair of electrons and π-electrons of quinone structures^28^. Carbohydrates and sugars present in the plant leaf extracts can also act as mild reducing agents^41^. Moreover, the carboxylate group present in the proteins which has greater affinity to act as surfactant by forming a protein layer on NPs could stabilize protein through negatively charged carboxylate group^26^. Further studies using advanced analytical tools, such as gas chromatography (GC) are required to determine the exact phytochemicals present in kaffir lime leaves, which are responsible for the bioreduction of silver.

### AgNPs generated hydroxyl radicals, but Ag + did not

In the cell free system or priming solution, only AgNPs solution generated •OH (Fig. 3), which correspond with previous reports using a cell free system^42^ and cell culture system^43^. In contrast, Ag^+^ derived from AgNO_3_ did not produce •OH in our test system, which is consistent with a previous study of Li *et al*.^42^ who reported that the addition of Ag^+^ inhibited the ESR signal or did not produce •OH in cell free system. Recent studies indicated that the formation of •OH by AgNPs occurs via a mechanism similar to Fenton reaction in which AgNPs act as a Fenton-like reagent^42, 43^ as follows:

Ag + H_2_O_2 _ + H^+^  = Ag^+^ + •OH + H_2_O

Since the valence of Ag changes from zero to one, the AgNPs may be viewed as “Fenton nanoparticles”, and the concentration dependence should be predicted by reaction^43^. That is, the production of •OH could be accelerated by increasing AgNPs or H_2_O_2_ concentration, or decreasing the pH of the buffer solution. Conversely, •OH production could be inhibited by addition of Ag^+^ or increasing the pH of the buffer^43^. Therefore, the presence of Ag^+^ as observed in our study could suppress the generation of •OH. The role of AgNPs mediated •OH generation for enhancing seed germination is discussed below.

### Nanopriming can stimulate seed germination of rice aged seeds by enhancing water uptake and starch metabolism

Seed priming with phytosynthesized AgNPs solutions at 10 and 20 mg L^-1^ induced more rapid water uptake at 4 and 24 h of imbibtion (Table 1). This data corresponded well with the early protrusion of radicles and higher germination percentage in nanoprimed seeds compared to other priming treatments and control. Previous literature highlighted that priming treatment can improve seed water uptake, as primed seeds exhibited a faster imbibition in comparison with non-primed ones, although pre-treated seeds were dried after priming to reach the same water content as non-primed ones^44^. Besides, seed priming with AuNPs also increased water uptake in maize plant^5^. Water uptake in seed germination process is essential because mature seeds are quite dry and need sufficient amount of water to start cellular metabolism and growth^45^. As the activities of dehydrogenase, a key enzyme in cellular respiration, in seedling roots of nanopriming treatments were higher than those of unprimed control and other treatments, this could be attributed to the consequence of rapid water uptake occurred in nanoprimed seeds. Thus, higher water uptake can enhance seed germination and seedling development through complex networks.

We also observed that two aquaporin genes, *PIP1;1* and *PIP2;11*, involved in water uptake were up-regulated in germinating seeds at 24 h of imbibitions (Fig. S5). Qian *et al*.^46^ also found that, in *Arabidopsis* seedlings, the transcript level of the aquaporins, including *PIP 1;2*, *PIP 2;1*, *PIP 2;2*, *SIP 1;1*, and *TIP 1;1* increased approximately 2-fold over the control levels after treatment with 0.2 or 0.5 mgL^−1^ AgNPs for one week. Aquaporins (water channels) are transmembrane proteins which not only facilitate the diffusion of water across biological membranes and regulate water homeostasis via a complex mechanism^46, 47^, but also facilitate gases such as CO_2_, nutrients, e.g., boron (B), silicon (Si), and reactive oxygen species (ROS)^47^. Previous studies indicated that certain concentrations of carbon nanotubes (CNTs) (40 μg mL^−1^) and AgNPs (0.2 and 0.5 mg L^−1^) can significantly activate the expression of aquaporin genes in tomato roots^48^ and *Arabidopsis* seedlings^46^, respectively. Besides, CNTs (multiwalled CNTs) can stimulate the expression of aquaporin genes and could play a role in the enhancement of seed water uptake during germination of some vegetable crops^49^. Thus, higher expression of aquaporin genes observed in nanoprimed seeds could be one of the mechanisms which enhanced seed germination.

We noted that at the time of water uptake or aquaporin gene expression increased, more H_2_O_2_ accumulation was also observed in nanoprimed seeds at 24 h of imbibition compared to unprimed and other primed seeds. This suggests that there is connection between ROS and aquaporin genes in controlling seed germination. Past studies suggested that a complex interplay may exist between aquaporins and biological relevant ROS^50^. Specific aquaporin isoforms can facilitate the passive diffusion of H_2_O_2_ across biological membranes and regulate the membrane permeability of H_2_O_2_, thereby acting in redox signaling process^50, 51^. Thus, the upregulation of aquaporin genes observed in this study could facilitate the diffusion of H_2_O_2_ in germinating rice seeds, which further lends support to our argument that both aquaporins and ROS could participate in enhancing seed germination (discussed later).

As demonstrated by colorimetric and starch-agar plate assays, we also found that nanopriming was the most effective treatment in improving starch metabolism of rice seeds and seedlings. Our results confirmed the existing literature that priming can enhance starch metabolism during germination^15, 52^. α-A mylase is hydrolytic enzyme responsible for the degradation of reserve carbohydrate to soluble sugars to maintain active respiratory metabolism for seed germination and subsequent seedling growth until photosynthesis is sufficient to support growth^9^. An increase in germination rate and seedling vigor of nanoprimed seeds observed in this study could partly be explained as a consequence of increased activity of α-amylase allowing faster rate of starch hydrolysis in germinating nanoprimed seeds. This resulted in more availability of soluble sugars necessary for generating the energy required for seedling growth, thereby leading to increased speed of germination.

We also found that nanopriming can enhance starch degradation during seed germination compared to control and other primed seeds (Fig. 6). Starch granules of nanoprimed seeds did not form the CSGs and contained some smaller ISGs, indicating higher degrees of starch decomposition. Moreover, some starch granules of nanoprimed seeds had small pits on their surface. A study by Man *et al*.^53^ found that α-amylase pitted the starch granule surface first, then penetrated into the interior and hydrolyzed the granule from the inside out, implying that α-amylase activity was induced higher in nanoprimed seeds during the first 24 h of imbibition. The increase in amylase activity and production in nanoprimed seeds resulted in higher starch degradation and rapid seed germination.

Earlier, Srivastava *et al*.^19^ suggested that pyrite NPs (FeS_2_ NPs) could produce H_2_O_2_ *in situ* on their surface in the presence of water, and are also considered as pyrite-only Fenton-like (PF) reagent, which could generate highly reactive •OH. These researchers also highlighted that FeS_2_ NPs could mimic the enzymatic activity of amylase enzyme to enhance the breakdown of starch in spinach seeds. Our study also revealed that AgNPs could act as Fenton like reagent and mediate the •OH generation as discussed above. Thus, AgNPs might behave in a similar manner to pyrite NPs in enhancing amylase activity in rice seed.

### Differential toxicity of Ag + (AgNO_3_) and AgNPs

Previous studies comparing the biological effects of AgNPs and AgNO_3_ have led to mixed conclusions; some studies revealed higher toxicity and/or bio-uptake for AgNPs than AgNO_3_^54, 55^; other studies report the opposite conclusion^56–58^. In the present study, Ag accumulation in seeds primed with AgNPs was lower than those of AgNO_3_ (see supporting information, Table S3). Similar results were also observed in *Allium* ^57^, castor^59^, and maize plants^58^, although treatment and culture methods of these studies were different from our approach. In addition, we also observed that seedlings developed from AgNO_3_ priming treatments showed stunted and slow growth compared to AgNPs priming treatments, indicating that AgNO_3_ showed phytotoxic effect than AgNPs. Our study confirmed the findings about stronger phytotoxic effect of AgNO_3_ compared to AgNPs previously reported by other authors^57–59^. Taken together, these data indicated that higher Ag accumulation could inhibit seed germination and seedling growth.

Although AgNO_3_ at appropriate concentrations, especially ultra-high dilution can be beneficial to plant growth^60^, silver ions at high concentration can cause phytotoxic effects in several plants^57–59^. It is known that Ag^+^ ions can interact metabolically with Cu and Se and replace H_2_ from the sulfhydryl groups of the photosynthetic enzymes, changing their structure and inactivating them. In addition, Ag can form complexes with amino acids, nitrogenous bases, and nucleotides, as well as with their corresponding macromolecular forms, indicating its potential to be either highly toxic or easily inactivated by the plant^61^. Besides, respiratory enzymes can be interfered by Ag^+^ ions due to their high affinity with thiol group of the enzymes^62^. Thus, higher concentration of Ag internalized into AgNO_3_-primed seeds can induce oxidative stress in seeds, leading to lower germination indices and seedling vigor index compared with AgNP-primed seeds.

It is known that the major factors that influence AgNPs toxicity and uptake in plants are attributed to the physico-chemical properties of particles, such as size, shape, surface coating, and experimental conditions including concentration, exposure time, method of exposure, and cell type or plant species^57, 63, 64^. Although the mechanism of toxic effects of AgNPs has not yet been clearly elucidated, it is generally believed that the toxicity of AgNPs on cells and organisms is partly driven by a release of Ag^+^ ions^57^. A previous study suggested that the oxidative stress effects of AgNPs were delayed as compared to AgNO_3_ due to the slower release of Ag^+^ ions from the AgNPs^65^. There was also evidence that Ag^+^ (released from AgNO_3_) exerted stronger toxicity on poplar and *Arabidopsis* growth than AgNPs^66^. In the present study, the release of Ag^+^ ions from AgNPs priming solution was relatively low (Table S2), corresponding with lower amount of Ag internalized into seeds (Table S3). Based on our data and past literature, it can be surmised that during the 24 h priming exposure, rice seed uptake of Ag^+^ from AgNPs was less than the AgNO_3_ due to slow release of AgNPs.

However, the effects of AgNPs are not simply due to the release of Ag^+^ ions into the surrounding environment as Ag^+^ and AgNPs commonly coexist^57^. It was suggested that AgNPs themselves can induce cellular oxidative stress and genotoxicity through overproduction of ROS^42^. Additionally, Li *et al*.^42^ demonstrated using ESR analysis that AgNPs can produce •OH directly, while AgNO_3_ did not. These data indicated that the mechanism of toxicity of AgNPs and Ag^+^ ions are different, and the nanoparticles, but not the released ions, mainly contribute to the AgNPs genotoxicity. ESR analysis also revealed that only AgNPs generated •OH. Thus, differential toxicity of AgNPs and Ag^+^ (AgNO_3_) could be due to the fact that AgNPs generate ROS slowly via their surface, while Ag + produces ROS via perturbation of the cell respiratory chain^42^.

As mentioned above, toxicity of AgNPs also depends on surface chemistry and coating. It has been reported that AgNPs synthesized using phytochemicals are coated by a thin layer of some capping organic material from the plant extracts and thus, this organic coating reduces their phytotoxicity than those synthesized using chemical methods^64^. Additionally, cellular uptake and variation of toxicity effects of NPs could be related to experimental conditions used, especially concentration and exposure time, and this would change (positive effect to negative or vice versa) if condition varies^63, 67^.

The concentrations of NPs used in past toxicological tests were extremely high and not conducted using a realistic environmental dose, which can lead to biased or misleading conclusion on the toxicity of these NPs, especially in low concentration ranges and limiting the potential beneficial applications of NPs in agriculture and other related technology^68^. Our study showed beneficial effects of AgNPs in term of nanopriming, in which condition used only low concentration of AgNPs and short exposure time, i.e. 24 h. This method not only reduces the toxicity of AgNPs, but also enhances the germination and starch metabolism of rice aged seeds. In addition, our ICP-OES analysis confirmed that no Ag uptake was observed in plant shoots and roots derived from seeds primed with nanopriming (Table S3). This implied that translocation of Ag element from seeds into plant vegetative organs did not occur or the Ag content in vegetative parts was below detection limit of the ICP-OES. This might be because the concentrations of AgNPs used in priming treatments were relatively low and seeds were exposed to the solution only for a short period. Thus, nanopriming would be alternative method for promoting seed germination for agricultural practices.

### Nanopriming modulated ROS-antioxidant enzyme system of aged seeds

Generally, seeds stored for a longer period are prone to oxidative damage due to the cumulative increase of ROS coupled with decline of the antioxidant potential of the cells, leading to seed aging and a loss of germination ability^69, 70^. In this study, we found that aged seeds without priming (unprimed seeds) showed lower activities of antioxidant enzymes, but higher levels of ROS, especially H_2_O_2_ at 0 h of imbibition (dry seed) (Fig. 7c,d). On the contrary, priming seeds with AgNPs can alter the activities of the antioxidant enzymes, especially CAT to higher levels, while reduce ROS production and accumulation after re-drying seeds (0 h of imbibition) compared to unprimed control and other treatments (Fig. 7b). As determined by spectrophotometric, *in vivo* fluorescence imaging, and histochemical localization assays, nanoprimed seeds produced higher H_2_O_2_ and O_2_^•–^ after 24 and 36 h of imbibition. Coincidently, antioxidant enzymes, i.e. SOD and CAT also increased significantly in nanoprimed seeds compared to unprimed and other primed seeds. Based on these evidences, therefore, alteration of ROS production and antioxidant enzyme activities in rice aged seeds after priming can be linked with higher germination in nanoprimed seeds compared with the unprimed ones.

In a recent concept of plant seed physiology, it has been proposed that the germination is completed only when the ROS content is within an oxidative window that allows ROS signaling^71, 72^. Above or below the ‘oxidative window for germination’, low or high amounts of ROS would not permit progress towards germination. Additionally, H_2_O_2_ is one of key ROS molecules acting as signalling molecule for the regulation of seed germination, and the precise regulation of H_2_O_2_ content by antioxidant machinery is essential to achieve a balance between oxidative signalling that stimulates germination and oxidative damage that prevents or delays germination^73^. Thus, higher level of H_2_O_2_ observed in nanoprimed seeds can act as signal molecule and was consistent with oxidative window concept, which resulted in better germination and faster seedling growth compared with that in unprimed and other primed seeds.

Antioxidant systems also play an important role in seed physiology^71^. In order to act as the positive regulator of germination, ROS accumulation must be tightly controlled by antioxidant systems. In the present study, activities of two antioxidant enzymes, i.e. SOD and CAT are relatively high in nanoprimed seeds at 24 h of imbibition, coinciding with high levels of ROS. Previous reports suggested that primed seeds exhibited more robust antioxidant system than unprimed ones during germination or early seedling establishment^8^, and increased antioxidant properties in primed seeds can enhance fitness of seedlings and plants subjected to stress conditions^52^. Similar to our finding, Kibinza *et al*.^74^ demonstrated that osmopriming sunflower seeds can increase the expression levels of a gene encoding catalase, and suggested that CAT is the key enzyme responsible for the recovery of vigor in aged seeds. SOD is the primary antioxidant enzyme that scavenges O_2_^•–^ to H_2_O_2_, while CAT catalyzes the conversion of H_2_O_2_ to H_2_O^75^. Considering the relationship between ROS and antioxidant enzymes, the high amount of antioxidant enzymes observed in nanoprimed seeds can be due to induction by AgNPs-mediated ROS, which is a tightly controlled mechanism to balance ROS in oxidative window range for stimulating seed germination as discuss above.

### Mechanism of enhancing seed germination and starch metabolism by nanopriming

To date, the mechanism underlying nanopriming-induced seed germination has not yet been reported in the literature. In the present study, the mechanism of beneficial roles of AgNPs in inducing seed germination was hypothetically proposed, including (i) creation of the seed coat nanopores, (ii) mild stress-induced agent or ROS-generating agent, and (iii) nanocatalyst for enhancing starch-degrading enzyme activity.

**Figure 11:**
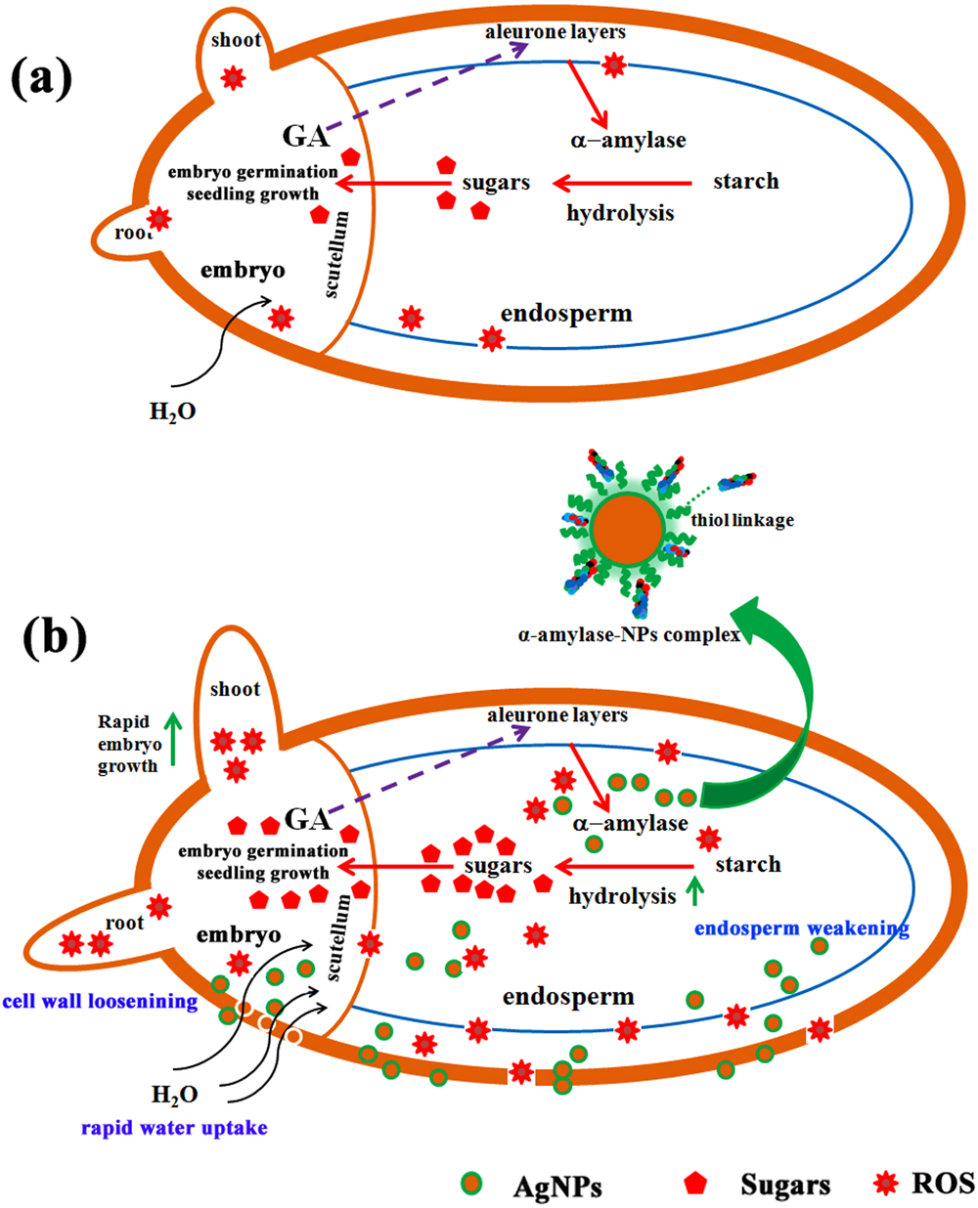
Proposed mechanism of AgNPs induced seed germination of rice. (**a**) Seed without AgNPs priming treatment has low metabolic activity due to slow water uptake; in this condition, the hydrolysis of starch occur gradually and thus available sugars is also low in the initial phase of imbibition, resulting in slow seed germination and seedling growth. (**b**) Seed priming with AgNPs can enhance seed germination with at least three possible scenarios. Firstly, AgNPs could penetrate seed coat by interacting with cell walls of seed coat to create small pores and thus enhance water uptake into seeds. Rapid water uptake can induce higher metabolic activity and starch hydrolysis of seed during initial phase of imbibition. Secondly, penetrated AgNPs could interact with α-amylase as NPs-amylase complex to fasten the starch hydrolysis and thus seed can generate higher available sugars to support embryo growth. Thirdly, AgNPs could mediate generation of ROS being in oxidative window range for acting as signalling molecules and participating in cell wall loosening and endosperm weakening. See texts for further explanation.

Although the precise mechanism behind nanoparticles action on physiological enhancement of seed germination is not clearly understood, one of the possible mechanisms to explain this scenario is based on Khodakovsakaya *et al*.’s hypothesis^76^. These authors hypothesized that carbon nanotubes were able to penetrate seed coat by creating small pores, resulting in increased water uptake and up-regulation of the expression of aquaporin genes involved water uptake. Our study also revealed that priming with AgNPs could enhance rapid water uptake and expression of aquaporin genes in germinating seeds. Meanwhile, a higher accumulation of ROS (H_2_O_2_ and O_2_^•–^) was observed in germinating seeds of nanopriming treatments than that of unprimed control and other priming treatments, indicating that more ROS are required for stimulating seed germination. Based on these evidences, both aquaporins and ROS could participate in activating seed germination. Previous studies suggested that aquaporins not only help water uptake, but also facilitate H_2_O_2_ or ROS diffusion across biological membranes^51^. Also, the interplay of aquaporins with ROS has been reported for regulating plant development^47, 50^. Thus, we further hypothesized that upon the formation of nanopores, the influx of the water into seeds becomes rapid, and also trigger the expression of aquaporin genes. Once aquaporin genes are up-regulated, they can facilitate the diffusion of ROS, especially H_2_O_2_ through biological membrane. The elevated ROS must be tightly controlled by seed antioxidant systems to produce ROS in optimum range of oxidative window as signalling molecules for triggering essential metabolic activity of seed for promoting seed germination and seedling development (Fig. 11).

Furthermore, we observed that amylase activity increased in response to nanopriming treatment resulting in increased soluble sugars. Increasing sugar concentrations in the cells can lead to lowering the osmotic potential, and hence water potential. This increases the difference (gradient) between the water potential outside and inside the tissues facilitating water movement into the seeds by osmosis. As soluble sugar contents increased in parallel to increased amylase activity in nanoprimed seeds, the enhancement of water uptake in their seeds could also be attributed to the change of internal osmotic potential due to increased soluble sugars (solutes).

Although plant cell growth is driven by water uptake, the growth is restricted by cell wall. As plant cell walls determine shape and the rate and direction of growth of individual cells as well as the mechanical resistance of whole tissues, the loosening of cell walls is an important process in all stages of plant development, requiring elongation growth or tissue weakening^77^. It has been approved that ROS, including •OH can act as a key component in cell wall loosening, testa and endosperm weakening, which is essential for radicle protrusion in seed germination^77–79^. As observed by ESR technique, AgNPs could mediate the generation of •OH, and thus act as ROS-generating agent. Hypothetically, it is likely that during the short duration (i.e. 24 h) of soaking seeds in nanopriming solutions, •OH generated by AgNPs could help loosening seed coat cell walls as well as endosperm weakening for enhancing seed germination. Besides, although ROS at high levels could induce oxidative stress, their optimum levels can be beneficial for seed germination. A slightly enhanced level of oxidative stress has been observed to stimulate seed germination^80^. Thus, priming seeds with AgNPs solutions could also induce mild oxidative stress for stimulating seed germination in addition to loosening cell wall.

We also propose another hypothesis that AgNPs penetrating the seeds might be important for their role as nanocatalyst in the hydrolysis of starch catalyzed by α-amylase and thereby increasing the reaction rate. It has been recently reported that AgNPs have the potential to act as nanocatalyst, and the rate of enzymatic digestion of soluble starch increased rapidly in the presence of nanosilver^81^. However, the exact mechanism behind the enhancement of starch hydrolysis by AgNPs is still unclear. A plausible explanation for one of the possible scenarios could be that, in the presence of AgNPs inside the seed, the α-amylase could possibly interact with functional groups of biomolecular ligands on the surface of phytochemicals-coated AgNPs through the thiol linkages, and thus the enzyme molecule is immobilized on the surface of AgNPs^81, 82^. Consequently, the α-amylase-bound nanoparticles complex (α-amylase-NPs complex) is able to enhance the rate of starch hydrolysis (Fig. 11). Structurally, α-amylase consists of two cysteine thiol groups which can expose to the medium or substrate and away from the active site^83^. It was suggested that AgNPs could be stabilized by the protein molecule through the thiol linkages and thus the enzyme molecule could be immobilized^81^. Therefore, it is possible that the α-amylase induced inside seeds could form the thiol linkages with functional groups of biomolecular ligands on the surface of phytochemicals-coated AgNPs with the active site being available for catalysis. The faster rate of starch hydrolysis of NP-amylase complex can be due to the enzyme conformational change, but the enzyme still retains its active site for catalysis^82, 83^. It has been well documented that upon a nanocarrier support, the efficiency of the enzyme increased compared to its free form^81, 84^. Therefore, the enhancement of enzyme activity and stability resulting in higher starch degradation may be attributed to the conformational change in the enzyme after interacting with molecules coated onto the surface of Ag nanocarriers. However, the exact mechanism warrants further investigation.

## Conclusion

In the present work, eco-friendly synthesis of AgNPs was successfully demonstrated through the use of kaffir lime leaves extracts as natural reducing and stabilizing agents. Various characterization techniques (UV-vis, TEM, EDX, SAED, XRD and FTIR) confirmed the formation of phytochemicals-capped AgNPs. We demonstrated that AgNPs can be applied as nanopriming agent for enhancing seed germination and starch metabolism of rice aged seeds. It was evident that AgNPs can internalize seed coat and support water uptake inside seeds, leading to promote seed germination and starch metabolism. Besides, our study indicated that priming with AgNPs priming could up-regulate the expression of aquaporin genes, thus facilitating water and H_2_O_2_ diffusion. Meanwhile, elevated ROS, especially H_2_O_2_ observed in nanoprimed seeds during germination could act as signal molecules for stimulating germination process. These evidences suggested that ROS and aquaporins play essential roles in enhancing seed germination. Furthermore, as evidenced by ESR technique, AgNPs could mediate the •OH generation, while AgNO3 did not. Thus, we hypothesized that when soaking seeds with nanopriming solution, •OH could help loosening seed coat cell wall and endosperms. Additionally, AgNPs penetrated into seeds, as determined by ICP-OES, could possibly interact with α-amylase enzyme or act as nanocatalyst; thereby enhancing seed starch degradation for seed germination and seedling growth. Since our nanopriming technique works with low concentration of AgNPs and does not apply NPs into soils, it prevents the dispersal of large amounts of NPs into the ecosystems. Nanopriming could be further exploited by seed companies as a new technique for commercial seed priming. Our study can serve as an initiative of nanopriming application for sustainable agricultural practices and agri-seed industry in the future.

## Methods

### Chemicals and materials used for AgNPs synthesis

AgNO_3_ (99.98%) was purchased from Merck (Germany). All aqueous solutions were prepared using double distilled deionized (DI) water. All reagents were of analytical grade. All glassware was washed with *aqua regia* solution and rinsed three times with DI water.

### Plant materials

Kaffir lime (*Citrus hystrix* D.C.), a small tree belonging to family Rutaceae, has been widely used as flavoring in Southeast Asian cuisines due to its highly aromatic and unique citrus flavors and strong fragrances. Kaffir lime leaves possess some important bioactive compounds (e.g. polyphenols), and given its good antioxidant properties^40^ we selected kaffir lime leaves (Inset of Figure S1 in Supporting information) as natural material source for AgNPs biosynthesis.

### Preparation of plant extract

Preparation of plant extract as reducing and stabilizing agents for AgNPs synthesis was done according to the method of Karthik *et al*.^37^ with some modifications. Briefly, the fresh leaf samples were initially rinsed with running tap water and then washed with DI water to remove the adhering dust particles and other contaminants. After blot drying, leaves (20 g) were cut into small pieces, blended in a kitchen blender with 100 ml of DI water. The blended extract was heated at 80 °C for 5 min and cooled at room temperature. Then, the extract was filtered through Whatman No.1 filter paper before centrifuging at 5000 rpm for 15 min to remove the heavy batteries. The filtered extract was kept in refrigerator (4 °C) up to 1 week for it to be used as reducing and stabilizing agents.

### Synthesis of silver nanoparticles (AgNPs)

In a typical reaction procedure, 3 mL of kaffir lime leaf extract was added to 12 mL of 1 mM AgNO_3_ aqueous solution and incubated in the dark for 1 h with gentle stirring. The solution turned to yellowish brown color after 1 h incubation and the reaction completed within 24 h. The AgNPs obtained from the solution were purified by repeated centrifugation at 10,000 g for 15 min followed by dispersion of the pellet in sterile DI water 4 times. The water-suspended nanoparticles were lyophilized for 48 h followed by characterization for the structure and composition.

### Characterization of phytosynthesized AgNPs

The bioreduction of the silver ions in the solution was determined under UV-visible spectroscopy using Perkin-Elmer Lambda 2 UV198 between 300 to 800 nm. Morphological details of purified AgNPs were measured with transmission electron microscopy (TEM) at 200 kV using FEI *TECNAI* G^2^ 20 equipped with selected area electron diffraction pattern (SAED). Simultaneously, the elemental compositions of the samples were also recorded using Energy Dispersive X-ray analysis. X-ray powder diffraction (XRD) using Cu *K*α radiation with *λ* = 0.15418 nm (Bruker D2 phaser) was used to investigate the crystalline nature and particles size of the synthesized AgNPs. Attenuated total reflectance Fourier transform infrared (ATR-FTIR) spectra of the freeze-dried samples were recorded using a Bruker Tensor 27 equipment to determine the surface functional groupsof the AgNPs.

### Preparation of priming solutions

The phytosynthesized AgNPs were used for seed priming test. AgNPs concentrations at 10 and 20 mg L^−1^, designated as AgNPs10 and AgNPs20 nanopriming agents, respectively, were freshly prepared by dispersing the particles in deionized water using ultrasonic vibration (100 w, 40 kHz) for 30 min. Similarly, for silver nitrate (AgNO_3_) priming solutions, two concentrations of AgNO_3_ at 10 and 20 mg L^−1^ designated as AgNO_3_10 and AgNO_3_20, respectively, were dissolved in deionized water and kept in dark bottle. Deionized water was used as hydropriming.

### Seed priming method

Seeds of jasmine rice (*Oryza sativa* L. cv. KDML105) were obtained from Khon Kaen Rice Research Centre, Thailand. These were naturally aged seeds due to long period of storage under ambient temperature (25–30 °C) for 3 years. The initial seed moisture content (MC) was 8.9% (on dry weight basis). Healthy seeds were selected from the same seed lot and used for all the experiments. For seed priming method, seeds were surface-sterilized in 3% H_2_O_2_ and then rinsed with deionized water for several times. Seeds were then soaked in AgNO_3_ or AgNPs priming solutions for 24 h with continuous aeration. The ratio of seed weight to solution volume was 1:4 (gmL^−1^). Seeds soaked in deionized water were defined as hydropriming (HP) and were washed three times with deionized water (3 min) and surface-dried on paper towel. Seeds were dried back to their original moisture content in shed at room temperature (25 ± 2 °C), sealed in polythene bags and stored at 4 °C until further use. The untreated seeds were used as the unprimed control (UP).

### Germination assay and growth measurement

Germination tests on the primed and unprimed seeds were performed in triplicates. Briefly, a piece of sterile filter paper (Whatman no.1) was placed on a sterile plastic Petri dish (90 × 15 mm) and moistened with 5.0 ml deionized water. Ten sterilized seeds were allowed to equilibrate under room temperature for 24 hrs, then placed in each plate, covered with lid and sealed with Parafilm M^®^ to avoid moisture loss. All Petri plates were kept in an incubator under dark at 25 °C.

After initiation of germination assay, seed germination was monitored daily for 6 days and seeds were considered germinated when the radicle was extended to more than 5 mm. Besides, at the end of germination experiment, length and weight of seedlings (roots and shoots) were measured. The data on germination percentage (GP), germination index (GI), germination rate (GR), and seedling vigour index (SVI) were calculated according to the equations provided elsewhere^85, 86^.

### Seed water uptake and dehydrogenase activity

The water uptake by seeds during imbibition at 4 and 24 h was determined following the method described elsewhere^87^. Dehydrogenase activity of the root tips was determined according to the method given by Singh *et al*.^88^

#### Starch metabolism and starch agar plate α-amylase assay

Starch metabolism in germinating seed and seedlings was assessed in terms of α-amylase activity and total soluble sugar content^15^. The α-amylase was determined following the method of Bernfeld^89^. Germinating seeds at 24 h of imbibition and seedling samples including shoot and root (1 g) were ground, mixed with 100 ml distilled water, and left standing for 24 h at 4 °C. The clear supernatant was collected and stored at −80 °C until analysis. The enzyme activity was determined from the supernatant by 3,5-dinitrosalicyclic acid (DNS) method^90^. In order to determine total soluble sugar contents, ground seedling sample (1.0 g) was mixed with 10 ml distilled water, left for 24 h at 25 °C^15^. The mixture was filtered with Whatman No. 42 and the final volume was made to 10 ml with distilled water. Total soluble sugar contents were determined by the phenol sulfuric method^91^.

The production of α-amylase using starch-agar plate assay was performed as described previously^32^. The embryoless half-seeds were surface sterilized with 1% sodium hypochlorite for 15 min and washed 5 times with sterilized water. The sterilized half-seeds were placed perpendicularly on autoclaved starch-agar plate [2% agar plate containing 0.2% soluble potato starch, 10 mM sodium acetate and 2 mM CaCl_2_ (pH 5.3)] and incubated at 25 °C in the dark for 3 days. GA plates were made by adding 1 μM gibberellic acid (GA_3_) to the cooled agar, while no GA_3_ was added to negative control plate. They were then removed and amylase production was examined by staining agar plate with IKI solution (0.1% I_2_ and 1% KI). Clear zones (transparent halos) appear if α-amylases are synthesized in endosperms and secreted into the starch agar, resulting in starch hydrolysis. Four endosperms of each treatment were placed in the same plate. All measurements were performed in triplicates.

### Starch granules morphology

After 24 h of imbibition, dehusked seeds from unprimed and primed groups (HP, SNP10 and SN10) were steeped in 0.2% NaOH for 2 days and homogenized in a blender. The homogenate was squeezed through five layers of cotton cloth and then filtered with 100-, 200-, and 400-mesh sieves, successively. Starch granules were washed and further purified according to a method described by Wei *et al*.^33^ The isolated starch granules were mounted on the specimen stub and sputter-coated with gold before viewing with scanning electron microscope (SEM, LEO 1450VP) at an accelerating voltage of 15 kV.

#### Measurement of reactive oxygen species and antioxidant enzyme activities

Hydrogen peroxide (H_2_O_2_) content in the rice seed embryos imbibed for 24 h was measured according to the method described by Patterson *et al*.^92^, while O_2_^•–^ content was determined according to the method of Elstner and Heupel^93^.

Antioxidant enzymes were extracted from aged rice embryos imbibed for 24 h. Seed embryos (~0.1 g) were homogenized in 2 mL of 50 mM potassium phosphate buffer (pH 7.8) containing 2 mM dithiothreitol (DDT), 0.1 mM ethylenediaminetetraacetic acid (EDTA), and 1.25 mM polyethylene glycol 4000 (PEG 4000) under cold conditions. Homogenate was centrifuged at 10,000 g for 15 min at 4 °C and the supernatant was used for the determination of superoxide dismutase (SOD, EC 1.15.1.1) and catalase (CAT, EC 1.11.1.6) as described elsewhere^70^.

### *in vivo* localization of hydrogen peroxide (H_2_O_2_) in germinating seeds

To *in vivo* localize and image H_2_O_2_ in germinating seeds, 2′,7′-dichlorofluorescein diacetate (DCF-DA) fluorescence probe was used to stain dehusked seeds after 24 h of imbibition according to the method of Bailly and Kranner^94^ with minor modifications. Briefly, imbibed seeds were stained with 25 µM DCF-DA in 20 mM potassium phosphate buffer (pH 6.0). Seeds were incubated at 25 °C in the dark for 30 min before rinsing three times with 20 mM potassium phosphate buffer (pH 6.0) for 5 min each. As negative control, seeds were preincubated with 5 mM ascorbate in darkness for 1 h at 25 ^o^C before adding the probe. The stained seeds were viewed at 4X on a Nikon ECLIPSE 80i fluorescence microscope with NIS-Element AR 3.2 sofware (Melville, NY, USA). The excitation and emission wavelengths for fluorescence probe are 488 and 525 nm, respectively.

### Histochemical localization of H_2_O_2_ and O_2_^•–^ in germinating rice seeds

To localize H_2_O_2_ and O_2_^•–^, DAB and NBT were used to stain seeds, respectively, as described by Chen *et al*.^95^ After 36 h of imbibition, five whole seeds were incubated with 1 mg mL^−1^ DAB solution (pH 3.8) or 1 mM NBT in 10 mM Tris-HCl (pH 7.0) at room temperature for 30 min. The stained seeds were washed with double-distilled water, and photographed through a bright field mode of Nikon ECLIPSE 80i fluorescence microscope.

### Determination of silver contents in seeds, seedlings and priming solutions

Unprimed and primed seeds (10 seeds from each replication) were dehusked and oven-dried at 80 °C for 48 h. Seeding shoots and roots (6 days old) were also oven-dried and used for Ag analysis. A representative sample of up to 0.5 g (seeds) or 0.1 g (shoots or roots) was digested in 9 mL of concentrated nitric acid and 3 mL of hydrofluoric acid for 15 min using the microwave heating system, diluted with MilliQ water and analyzed using inductively coupled plasma atomic emission spectroscopy (Optima 2100 DV ICP-OES, Perkin Elmer Instruments, USA) as described elsewhere^96^. Similarly, Ag^+^ ions in priming solutions, i.e. AgNPs10, AgNPs20, AgNO_3_10, AgNO_3_20, and DI water were also analyzed using ICP-OES.

### Electron Spin Resonance (ESR) analysis of hydroxyl radicals

The ESR analysis of hydroxyl radicals (•OH) was carried out according to the method of Li *et al*.^42^, except that after addition of AgNPs or AgNO_3_ to spin trap, the particle materials were removed by centrifugation and filtration according to the recommendation of Jeong *et al*.^97^ All ESR measurements were performed at room temperature using a Bruker Elexys500 spectrometer. The ESR parameters were as follows: 1 G field modulation, 100 G scan range and 20 mW microwave power for detection of spin adducts using 5-dimethyl-pyrroline *N*-oxide (DMPO) spin trap (Santa Cruz Biotechnology).

### RNA extraction and gene expression analysis

Total RNA was extracted from fresh rice embryos imbibed for 24 h using the PureLink^®^ RNA Mini Kit (Life Technologies, USA) according to the manufacturer’s instructions. Isolated RNA was further purified using the PureLink^TM^ DNase Set (Invitrogen, USA) following the manufacturer’s protocol. The purity and concentration of RNA were checked using the NANOdrop spectrophotometer. First strand cDNA synthesis was performed using SuperScript^®^ III First-Strand Synthesis System for RT-PCR (Invitrogen, USA) according to the manufacturer’s instructions. Expression analysis of the two aquaporin genes using semi-quantitative reverse transcriptase-polymerase chain reaction (sqRT-PCR) was essentially as described previously^98^. Primer sequences for the studied aquaporin and actin genes were designed according to Sakurai *et al*.^98^ and Nounjan *et al*.^99^, respectively.

### Statistical analysis

A one-way analysis of variance (ANOVA) was performed between treatment samples in a completely randomized block design using three replications and data were analyzed using IMB SPSS statistics version 22. The significant levels of difference for all measured traits were calculated and means were compared by Duncan’s Multiple Range Test at 5% probability level. The *p* value < 0.05 was considered as statistically significant.

## Electronic supplementary material

Supplementary Information
