# Supplementary material for: Nanopriming technology for enhancing germination and starch metabolism of aged rice seeds using phytosynthesized silver nanoparticles
Source: Sci Rep. 2017 Aug 15;7:8263. doi: 10.1038/s41598-017-08669-5 (PMC5557806; doi:10.1038/s41598-017-08669-5)
Supplement: Supplementary file 1 — Supplementary Information [file 41598_2017_8669_MOESM1_ESM.doc]

**Supporting Information**

**Nanopriming technology for enhancing germination and starch metabolism of aged rice seeds using phytosynthesized silver nanoparticles**

Wuttipong Mahakham^1^, Ajit K. Sarmah^1*^ Santi Maensiri^2^, Piyada Theerakulpisut^3*^

^1^Department of Civil and Environmental Engineering, Faculty of Engineering,

The University of Auckland, Private Bag 92019, Auckland 1142, New Zealand

^2^School of Physics, Institute of Science, Suranaree University of Technology,

Nakhon Ratchasima 30000, Thailand

^3^Salt-tolerant Rice Research Group, Department of Biology, Faculty of Science,

Khon Kaen University, Khon Kaen 40002, Thailand

***Corresponding authors**

[a.sarmah@auckland.ac.nz](mailto:a.sarmah@auckland.ac.nz) (AS), [piythe@kku.ac.th](mailto:piythe@kku.ac.th) (PT)

**Figure captions**

**Fig.S1.** UV–vis spectral range from 300 to 700 nm showing a peak at 443 nm; insert shows kaffir lime leaves, the plant extract, AgNO_3_, and AgNPs solution, respectively. (For interpretation of the references to color in this figure legend, the reader is referred to the web version of this article.)

**Fig.S2.** EDX spectrum of AgNPs synthesized using kaffir lime leaf extract

**Fig.S3.** Effect of different priming treatments on root length (a), shoot length (b), seedling length (c) and seedling biomass (d). Data are presented as means ± SE. Different letters above bars denote statistical difference after one way ANOVA test at *p ≤* 0.05.

**Fig.S4.** Starch agar plate assay for detecting α-amylase production of different primed seeds compared with unprimed control. Seeds imbibed for 24 h before cutting as embryoless half-seeds. After placing the half seeds in starch agar plate at 25 °C for 3 days, plates were stained with IKI solution. Clear zone indicates amylase production, (1) unprimed seeds, (2) hydroprimed seeds, (3) AgNO_3_10-primed seeds, (4) AgNO_3_20-primed seeds, (5) AgNPs10-primed seeds, and (6) AgNPs20-primed seeds.

**Fig.S5.** Expression of two aquaporin genes, *PIP1;1* and *PIP2;1* rice embryos of different priming treatments showing differential expression after 24 h of seed imbibitions. (1) (1) unprimed seeds, (2) hydroprimed seeds, (3) AgNO_3_10-primed seeds, (4) AgNO_3_20-primed seeds, (5) AgNPs10-primed seeds, and (6) AgNPs20-primed seeds.


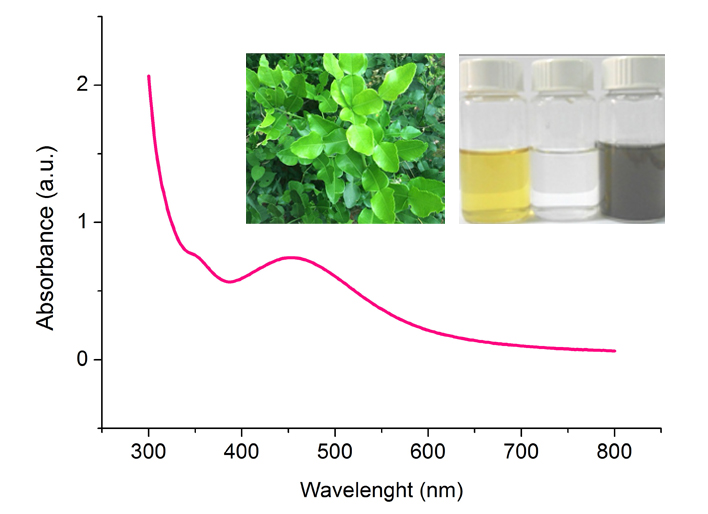


**Fig. S1.**


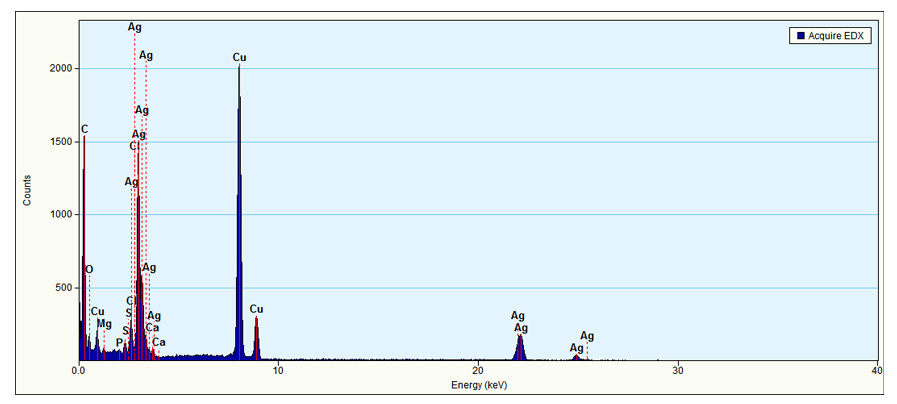


**Fig. S2.**

**
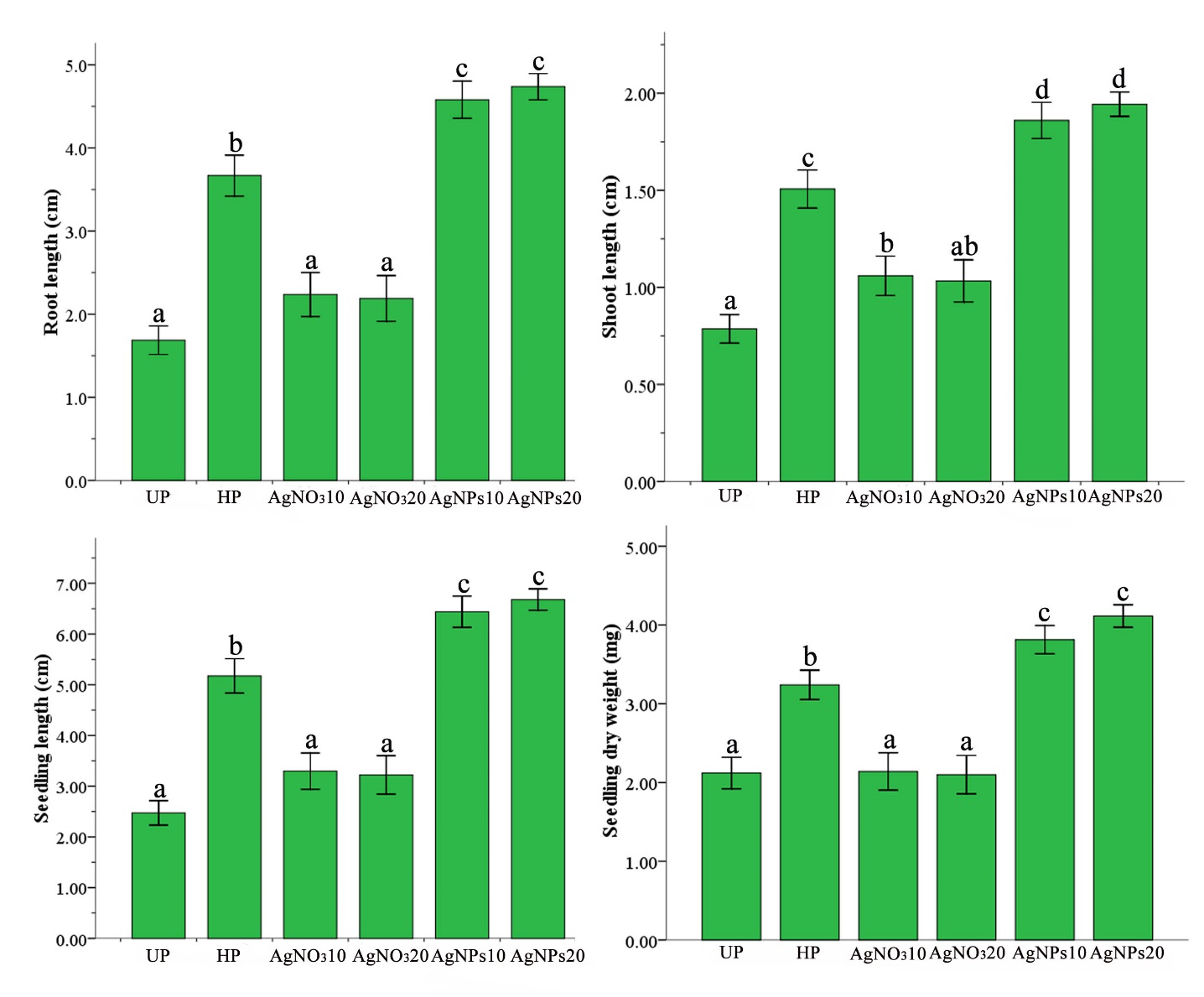
**

**Fig.S3.**


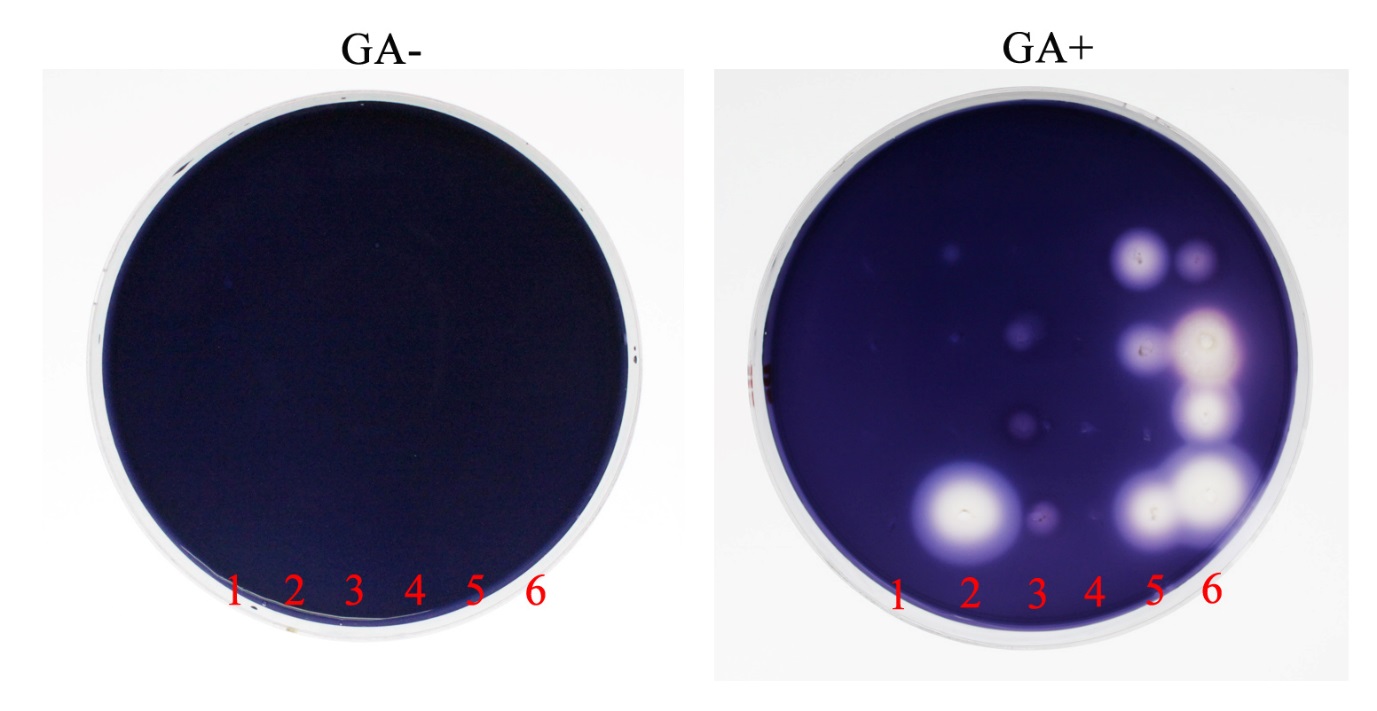


**Fig.S4**


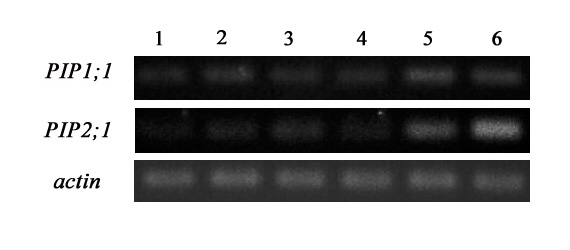


**Fig.S5**

**Table S1.** Calculation of the average particle size of AgNPs synthesized by kaffir lime leaf extracts by using Debye Scherer’s equation.

| S. No | 2θ (degree) | FWHM (β) (radians) | d-spacing (ºA) | Miller indices | Crystallite size (nm) |
| --- | --- | --- | --- | --- | --- |
| 1. | 38.2 | 0.634 | 2.373 | [111] | 13.3 |
| 2. | 44.3 | 1.156 | 2.096 | [200] | 7.4 |
| 3. | 64.6 | 0.749 | 1.445 | [220] | 12.5 |
| 4. | 77.4 | 0.887 | 1.237 | [311] | 11.5  Average size (D) = 11.2 |

**Table S2.** Ag^+^ ions releases from different priming solutions at 1 h and 24 h after dissolution determined by ICP-OES technique. Values are means of three replicates ± standard deviation; means with different letters are statistically different (Duncan’s multiple comparison at *p ≤* 0.05).

| Priming solutions | Ag^+^ concentrations (mg L^-1^) | |
| --- | --- | --- |
|  | 1 h | 24 h |
| AgNO_3_10 | 0.97d | 6.5c |
| AgNO_3_20 | 11.63b | 13.05a |
| AgNPs10 | 0.19af | 0.30ef |
| AgNPs20 | 0.43ef | 0.51e |
| Deionized water | N.D.* | N.D.* |

* value was below detection limit of ICP-OES analysis (0.001 ppm for Ag).

**Table S3.** Silver concentrations (mg/kg DW biomass) in rice seed endosperms. Endosperms of unprimed (UP) and primed seeds were collected after 24 h germination for ICP-OES analysis. For shoots and roots, 6-day old seedlings were used for measuring Ag concentrations. Values are means of three replicates ± standard deviation; means with different letters are statistically different (Duncan’s multiple comparison at *p ≤* 0.05).

| plant tissues | UP | HP | AgNO_3_10 | AgNO_3_20 | AgNPs10 | AgNPs20 |
| --- | --- | --- | --- | --- | --- | --- |
| seeds (endosperm) | N.D.* | N.D.* | 0.65 ± 0.26b | 0.89 ± 0.11c | 0.46 ± 0.12a | 0.57 ± 0.01ab |
| shoots | N.D.* | N.D.* | N.D.* | N.D.* | N.D.* | N.D.* |
| roots | N.D.* | N.D.* | N.D.* | N.D.* | N.D.* | N.D.* |

* value was below detection limit of ICP-OES analysis (0.001 ppm for Ag).
